# Discovery of a fragment hit compound targeting D-Ala:D-Ala ligase of bacterial peptidoglycan biosynthesis

**DOI:** 10.1080/14756366.2022.2149745

**Published:** 2022-11-29

**Authors:** Matic Proj, Martina Hrast, Gregor Bajc, Rok Frlan, Anže Meden, Matej Butala, Stanislav Gobec

**Affiliations:** a Faculty of Pharmacy, Department of Pharmaceutical Chemistry, University of Ljubljana, Ljubljana, Slovenia; b Biotechnical Faculty, Department of Biology, University of Ljubljana, Ljubljana, Slovenia

**Keywords:** Fragment-based drug discovery, hit triage, inhibitors, antibacterial agents

## Abstract

Bacterial resistance is an increasing threat to healthcare systems, highlighting the need for discovering new antibacterial agents. An established technique, fragment-based drug discovery, was used to target a bacterial enzyme Ddl involved in the biosynthesis of peptidoglycan. We assembled general and focused fragment libraries that were screened in a biochemical inhibition assay. Screening revealed a new fragment-hit inhibitor of DdlB with a Ki value of 20.7 ± 4.5 µM. Binding to the enzyme was confirmed by an orthogonal biophysical method, surface plasmon resonance, making the hit a promising starting point for fragment development.

## Graphical Abstract

**Figure:**
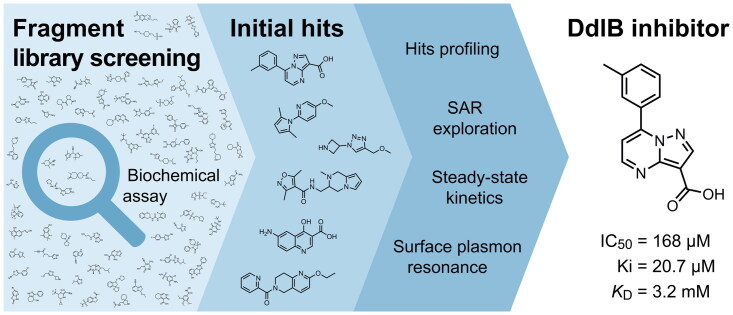

## Introduction

Infections caused by antibiotic-resistant bacteria pose a serious challenge to healthcare systems worldwide. The increasing resistance of gram-positive and gram-negative pathogens causing infections in hospitals and in the general population and the worldwide spread of antibiotic-resistant 'superbugs’ represent a major global health problem^1^. Reportedly, 25,000 Europeans die each year as a direct result of infections with multidrug-resistant strains of pathogenic bacteria, with an estimated economic impact of 1.5€ billion per year^2^. However, the pipeline for new drugs is small because major pharmaceutical companies have largely abandoned antibiotic research, despite the urgent need for new drugs^3^. The fact that few new antimicrobials are available and multidrug resistance is becoming more common means that we need to increase our efforts in finding new antimicrobials^4^. It is imperative that we continue to search for new antibacterial agents by using innovative screening methods for carefully selected protein targets and by conducting rational drug design using the advances offered by protein crystal structures.

**Figure 1.**
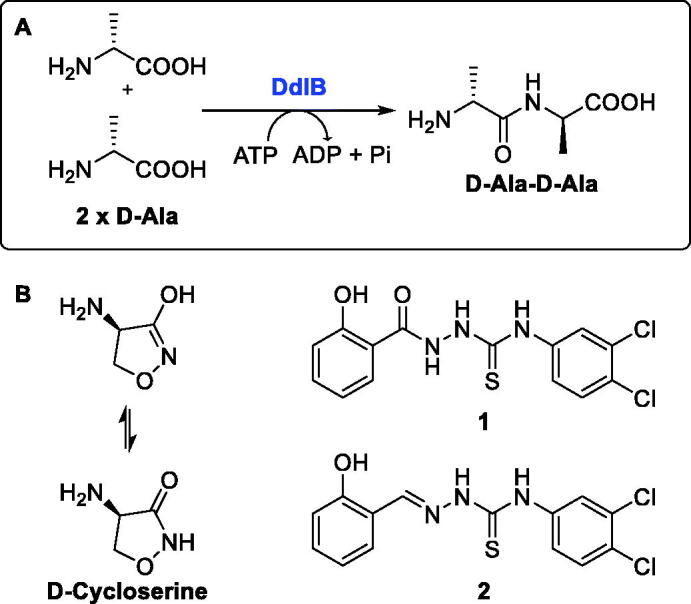
(**A**) Reaction mechanism and (**B**) inhibitors of Ddl.

Peptidoglycan is a macromolecule essential for bacterial survival and is found only in the bacterial cell wall. Therefore, enzymes involved in the peptidoglycan biosynthetic pathway represent potential targets for the discovery of new antimicrobial agents^5^^,^^6^. Among the intracellular enzymes involved in peptidoglycan biosynthesis, only two enzymes have been validated as antibacterial targets by inhibitors that are in clinical use: UDP-*N*-acetylglucosamine-enolpyruvyl transferase (MurA, EC 2.5.1.7) is validated by fosfomycin, which is used to treat urinary tract infections^7^, and D-alanine:D-alanine ligase (Ddl, EC 6.3.2.4) is validated by cycloserine (Figure 1(B)), which is a second-line drug for the treatment of tuberculosis^8^.

Ddl is an ATP-dependent bacterial enzyme that catalyses the ligation of two D-alanines to a D-alanyl-D-alanine product (Figure 1(A)), which in the next step is incorporated into the final intracellular peptidoglycan precursor UDP-*N*-acetylmuramoyl pentapeptide^5^. Subsequently, this D-alanyl-D-alanine terminus is involved in transpeptidation, the cross-linking of the growing peptidoglycan chains^9^. In *Escherichia coli*, there are two isozymes of Ddl, DdlA and DdlB, which have similar catalytic efficiencies and substrate recognition properties, as well as similar sensitivity to inhibitors^10^. We focussed on the search for inhibitors of DdlB because it is the best studied and the crystal structures are available^11^. Although many Ddl inhibitors have been discovered in the past two decades, mainly by various screening campaigns or by classical medicinal chemistry approaches (for a comprehensive review, see ref.^12^), these inhibitors usually had only weak or no antibacterial activity. The most promising recent inhibitors appear to be thiosemicarbazide **1** and thiosemicarbazone **2** (Figure 1(B)), which had promising antibacterial activity and have been shown to target Ddl in bacteria^13^^,^^14^.

One of the innovative and underutilised approaches to target Ddl is fragment-based drug discovery (FBDD). This approach is increasingly being used in the pharmaceutical industry and academia to reduce attrition and provide leads for previously inaccessible biological targets. FBDD identifies low molecular weight ligands (∼150 Da) that bind to biologically important macromolecules. The three-dimensional experimental-binding mode of these fragments is determined by X-ray crystallography or NMR spectroscopy and is used to facilitate their optimisation into potent lead compounds with drug-like properties by known methods such as fragment growing and fragment linking. Compared with high-throughput screening, the fragment-based approach requires a smaller number of compounds to be screened and provides more efficient and fruitful optimisation campaigns^15^^,^^16^. In this manuscript, we describe the construction of a new library of fragments and its screening on DdlB from *E. coli* to discover new fragment-hit inhibitors.

## Results and discussion

The definition of fragments in this study was based on physicochemical properties, the most important criterion being a molecular weight not exceeding 300 Da. Half of the fragment library was designed for a general purpose and will be screened on other targets as well. It contained diverse fragments (purchased or available in-house) without reactive functional groups. The second half consisted of subsets specifically designed to target DdlB. Since the enzyme is a dinuclear Mg^2+^-dependent metalloenzyme, fragments capable of chelating metal ions could potentially bind to the active site via coordination interactions with Mg^2+^ ions. A subset of chelating fragments was designed using substructure filters for chelating groups (Supplementary Table S2)^17^^,^^18^. DdlB is also ATP-dependent, thus we designed a subset of fragments containing phosphate bioisosteres (Supplementary Table S3) aiming to bind into the phosphate-binding site pockets normally occupied by ATP^19^. Next, we designed a subset of cycloserine analogues that have similar substructures to an approved drug cycloserine and could potentially bind to the D-Ala-binding site (Supplementary Table S4). The final subset was designed by selecting the highest scoring hits obtained by docking a virtual library of available fragments to ATP- and D-Ala-binding sites. In total, the fragment library contained 943 compounds (Table 1) distributed across a range of physicochemical properties (Supplementary Figure S1).

**Table 1.** Fragment library subsets and number of fragments used for screening on DdlB.

Fragment library subsets	Number of fragments
In-house diverse fragments	195
Purchased diverse fragments	283
Chelating fragments	214
Phosphate bioisostere fragments	159
Cycloserine analogues	31
Docked fragments to DdlB	61
Total	943

The fragment library was assayed in a DdlB inhibition assay, where fragments were tested for their ability to inhibit the D-Ala-adding activity of DdlB ligase. The orthophosphate formed during the enzymatic reaction was measured spectrophotometrically using malachite green reagent. The tested fragment concentrations varied from 1.5 to 5 mM. The criteria for classifying a fragment as a hit were: <50% residual DdlB activity and solubility (low background absorbance of the compound at 650 nm). The results are provided in a Supplementary excel file. We re-purchased six hit compounds that matched the criteria to determine the inhibition of DdlB from freshly prepared compound solutions (Table 2). However, only two hits showed sufficient inhibition for IC_50_ determination (**3** and **4**).

**Table 2.** Profiling of repurchased fragment hits from the screening.

Structure	Label	Fragment library subset	DdlB inhibition	MurA inhibition	Phosphate binding	HRP-PR assay	H_2_DCFDA assay	Resazurin assay	Thiol reactivity
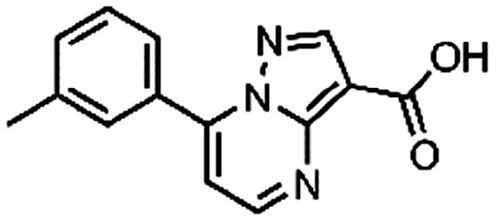	**3**	Docked fragments to DdlB	IC_50_ = 168 μM	64% RA at 5 mM	111% free phosphate at 5 mM	Not active	Not active	Not active	Not reactive
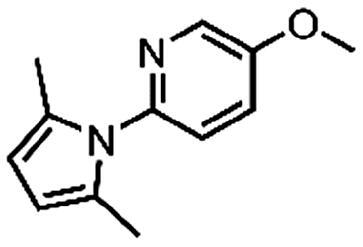	**4**	Purchased diverse fragments	IC_50_ = 1790 μM	39% RA at 5 mM	29% free phosphate at 5 mM	Not active	Active	Active	Reactive
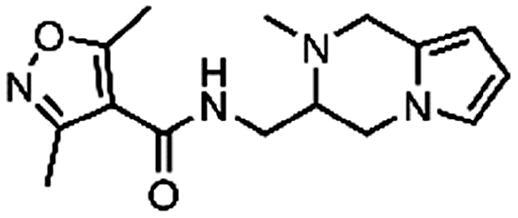	**5**	Purchased diverse fragments	28% RA at 2.5 mM	6% RA at 5 mM	10% free phosphate at 5 mM	Not active	Active	Not active	Not reactive
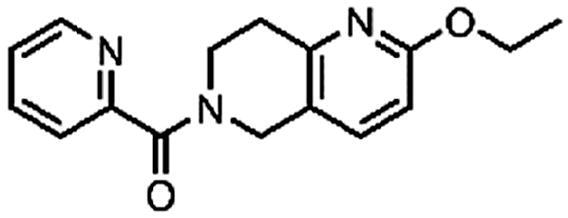	**6**	Purchased diverse fragments	25% RA at 5 mM	104% RA at 5 mM	23% free phosphate at 5 mM	Not active	Not active	Active	Not reactive
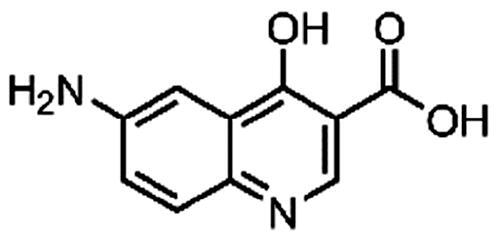	**7**	Docked fragments to DdlB	47% RA at 5 mM	81% RA at 5 mM	89% free phosphate at 5 mM	Not active	Not active	Not active	Not reactive
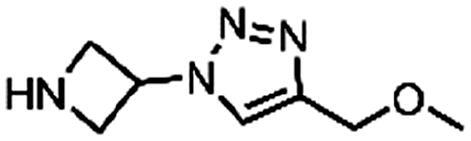	**8**	Purchased diverse fragments	95% RA at 5 mM	100% RA at 5 mM	85% free phosphate at 5 mM	Not active	Active	Not active	Not reactive

RA: Residual Activity.

To pursue only compounds with tractable mechanism of action, extensive profiling of fragment hits was performed. First, deselection was performed with a MurA inhibition assay using the same assay technology to find compounds that interfered with the malachite green assay system. Second, the fragment hits were tested in a phosphate-binding assay that omitted the enzyme and again used the same assay technology. Compounds **4** and **5** were found to interfere with the assay by inhibiting MurA, DdlB, and binding phosphate (Table 2). Redox activity assays were performed to find compounds that interfered with the assay or had undesirable mechanisms of bioreactivity leading to false positive inhibitions. The horseradish peroxidase-phenol red (HRP-PR) assay was used to detect H_2_O_2_ generated by redox cycling compounds in the presence or absence of DTT. A fluorescent probe, 2′,7′-dichlorodihydrofluorescein diacetate (H_2_DCFDA), was used to detect compounds generating reactive oxygen species in the presence or absence of a reducing agent TCEP. The resazurin assay was used to detect redox active compounds that catalyse the conversion of resazurin to resorufin by the formation of free radicals in the presence of the reducing agent DTT. Compounds **4**–**6** and **8** were found to be active in at least one of the redox activity assays (Table 2, Table S5). Because our goal was to discover noncovalently binding inhibitors of DdlB, the fragment hits were assayed for their potential reactivity with a nucleophilic thiol surrogate, reduced 5,5-dithio-bis-(2-nitrobenzoic acid) (DTNB). Fragment **4** was found to be reactive in this assay (Table 2 and Supplementary Figure S2).

**Figure 2.**
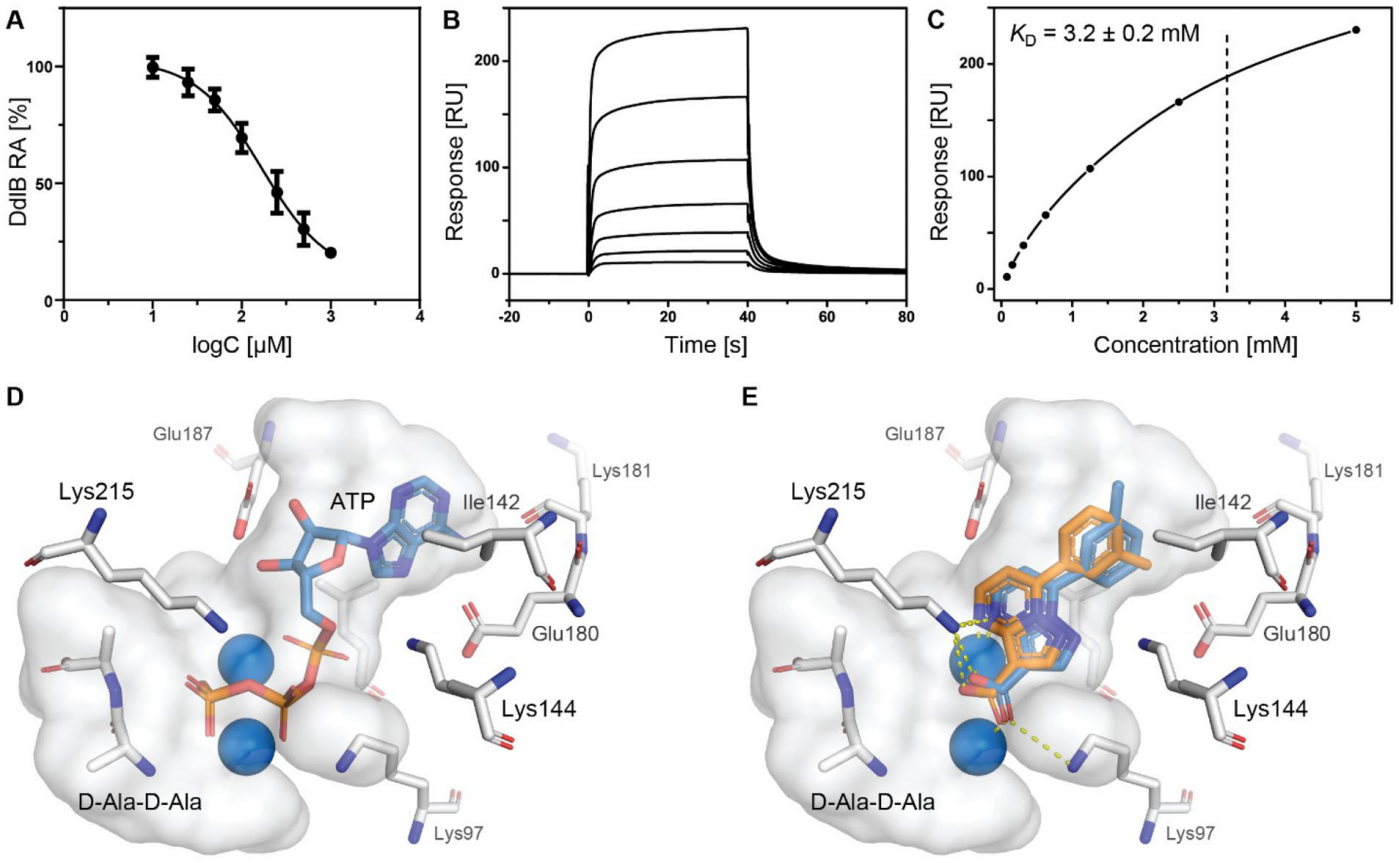
Fragment hit **3** targeting DdlB. (**A**) Dose–response inhibition of DdlB for **3**. (**B**) SPR analysis of **3** with the immobilised DdlB. **3** was injected across DdlB in serial dilutions ranging from 78.125 to 5000 µM for 40 s at a flow rate of 30 μL/min, and dissociation was followed for 40 s. Three independent measurements were performed and representative sensorgrams revealing dose-dependent binding of **3** to DdlB are shown. (**C**) Data were fitted to the steady-state affinity model to obtain the apparent equilibrium dissociation constant (*K*_D_). *K*_D_ values are the mean ± standard deviation of three titrations. (**D**) ATP (blue sticks) and D-Ala-D-Ala (white sticks) binding sites of DdlB (PDB ID: 4C5C) with the co-crystallised ligands. (**E**) Computational docking of **3** to the ATP-binding site using extra-precision Glide (blue sticks) and QM-Polarized Ligand Docking (orange sticks) (PDB ID: 4C5C).

Based on the fragment hit profiling results, compounds with at least two red flags were excluded. For further evaluation, we selected fragment hit **3** without red flags and with an IC_50_ value of 168 μM (Figure 2(A) and Table 2), which is in the same range as that of the approved drug cycloserine (IC_50_ = 108 μM). Compound **3** was part of a *Docked fragments to DdlB* subset of the fragment library. In particular, docking to the ATP-binding site was used in the selection of this compound as part of the fragment library design. Structurally, **3** is a pyrazolopyrimidinecarboxylic acid with a molecular weight of 253 Da.

In an attempt to investigate the structure-activity relationship, 15 analogues of **3** were purchased (analogue-by-catalogue) (Table 3). Compounds **9**–**14** differ by the substituents on the phenyl ring, while other heterocycles were explored with compounds **15**–**18**. The carboxylic group was switched from position 3 to position 2 for compounds **18** and **19**. Compounds **20**–**23** contain isosteric replacements for the carboxylic group. None of the assayed analogues inhibited DdlB in an inhibition assay. However, because they are fragment-sized compounds (molecular weight between 234 and 322 Da), even small changes may contribute to loss of inhibitory potency^20^.

**Table 3.** Analogues of compound **3** that were evaluated in the DdlB inhibition assay.

Structure	Label	DdlB inhibition
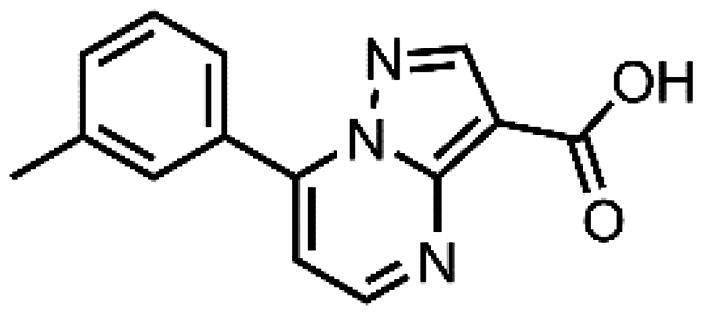	**3**	IC_50_ = 168 μM Ki = 20.7 µM
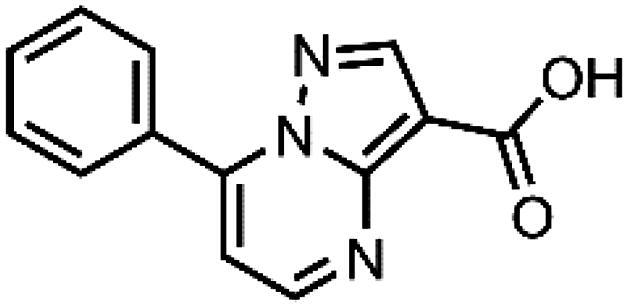	**9**	90% RA at 5 mM
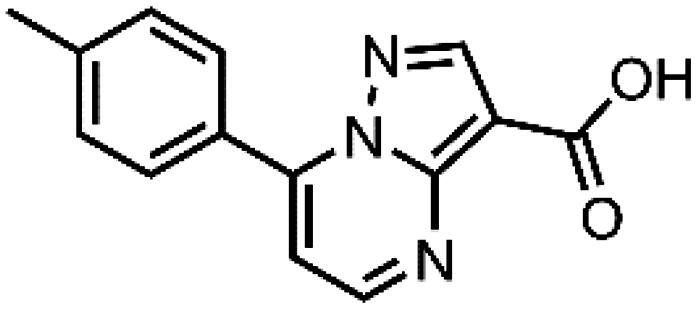	**10**	101% RA at 0.5 mM
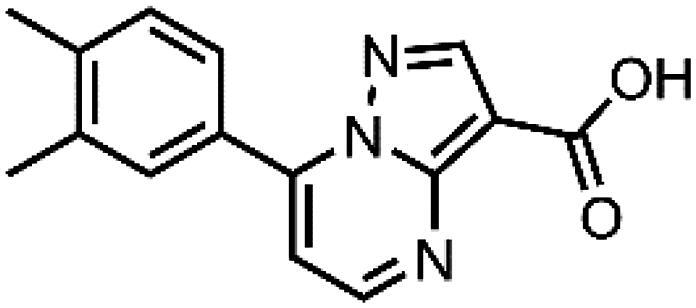	**11**	100% RA at 0.5 mM
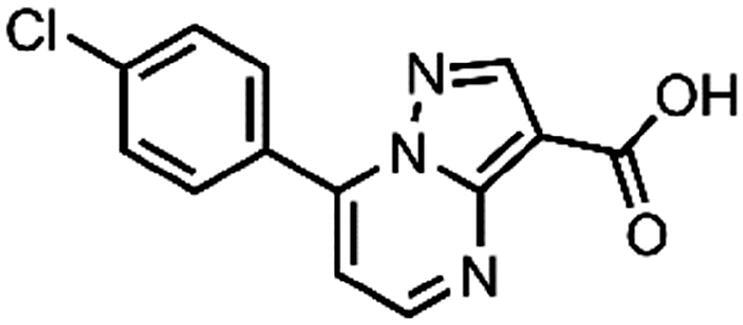	**12**	0% RA at 5 mM 90% RA at 1 mM
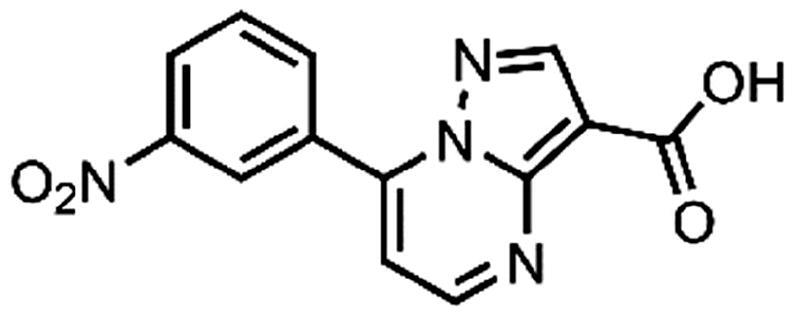	**13**	89% RA at 0.5 mM
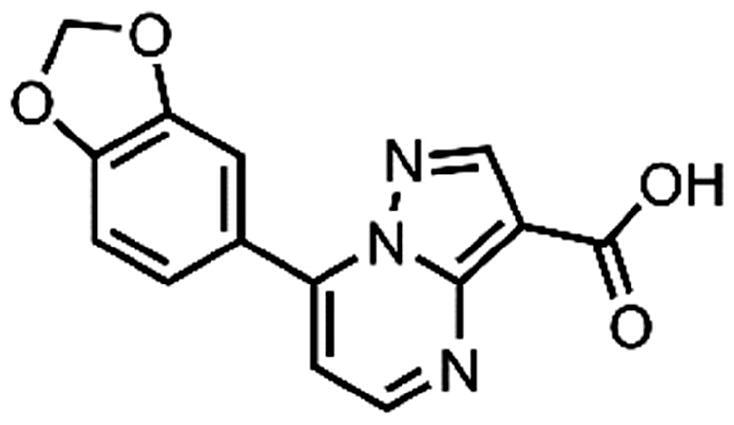	**14**	100% RA at 0.5 mM
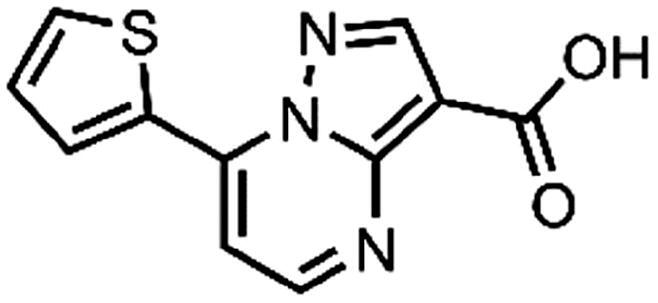	**15**	96% RA at 1 mM
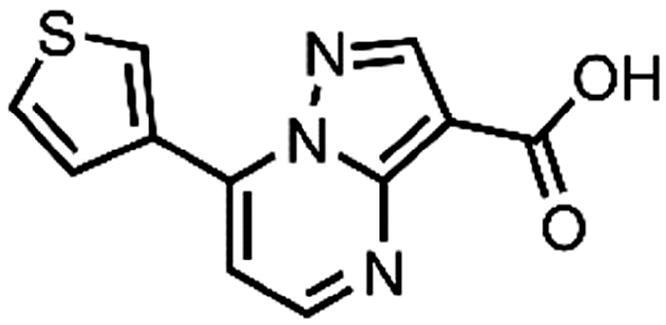	**16**	89% RA at 0.5 mM
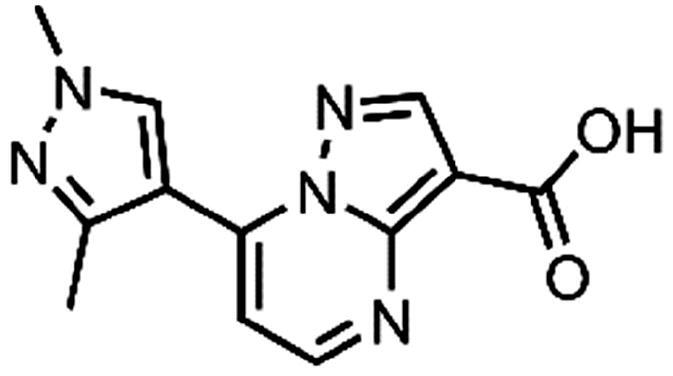	**17**	26% RA at 5 mM Nonsigmoidal curve
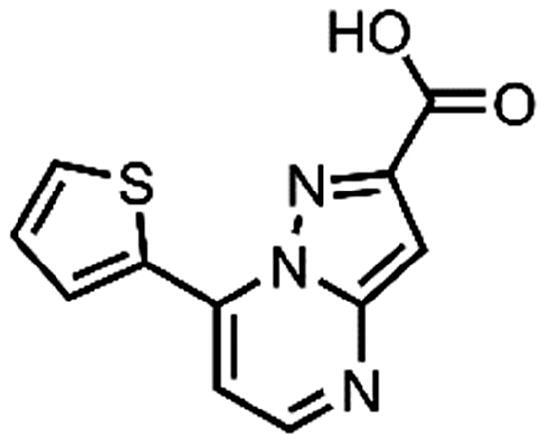	**18**	69% RA at 5 mM 87% RA at 1.5 mM
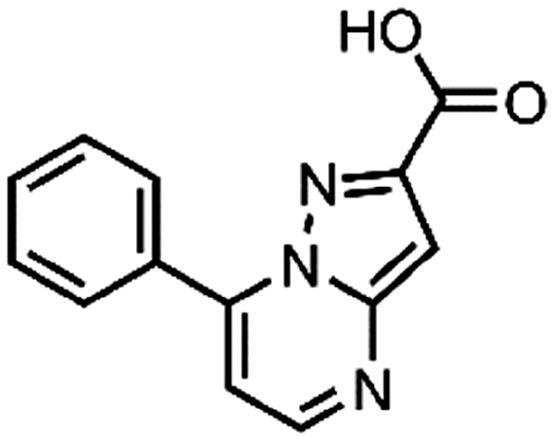	**19**	50% RA at 5 mM 90% RA at 1.5 mM
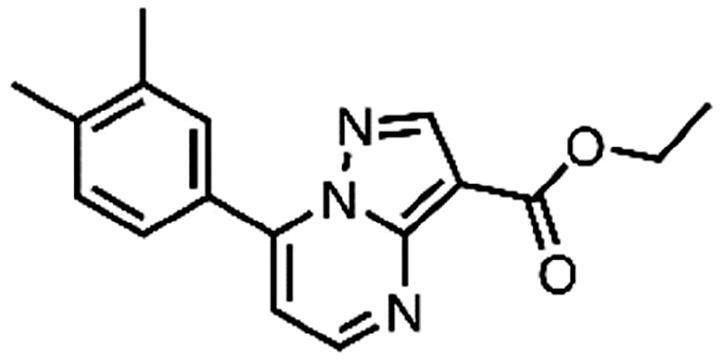	**20**	100% RA at 0.5 mM
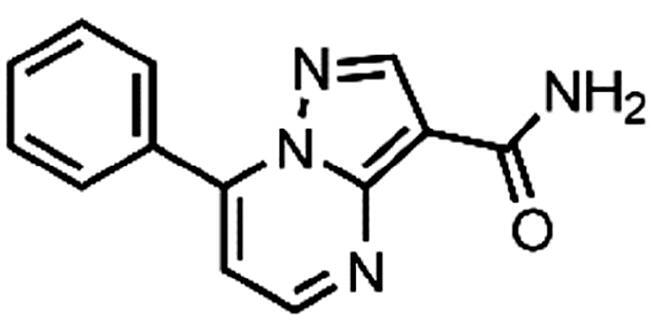	**21**	96% RA at 0.5 mM
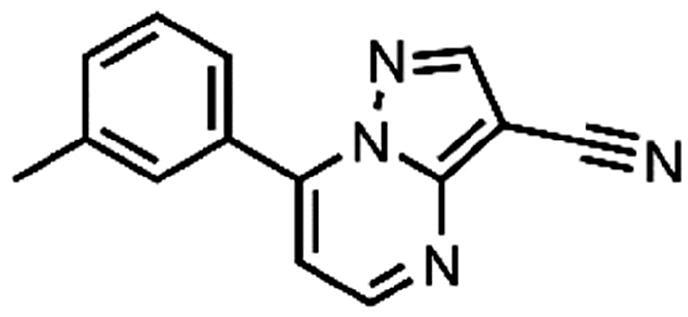	**22**	100% RA at 0.5 mM
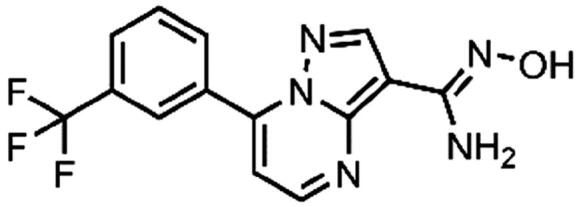	**23**	94% RA at 0.5 mM
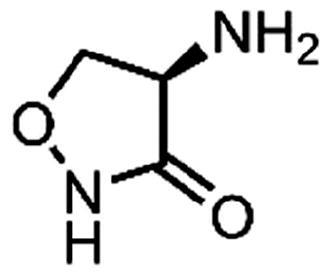	**D-Cyclo-serine**	IC_50_ = 108 μM

Binding of **3** to DdlB was confirmed by an orthogonal biophysical assay, surface plasmon resonance (SPR). The protein was covalently attached to the surface of the CM5 chip (∼7700 response units), and the ligand was titrated across the surface (Figure 2(B)). The apparent equilibrium dissociation constant (*K*_D_ = 3.2 mM) was estimated from the steady-state binding levels (Figure 2(C)). Steady-state kinetics were performed to gain mechanistic insight into the inhibition of compound **3** (Supplementary Figure S3 and Table S6). The resulting best model showed that **3** is a competitive inhibitor with respect to ATP with a Ki value of 20.7 ± 4.5 µM. These data are in agreement with the docking results obtained during the design of fragment library, where **3** was identified as a potential ATP competitive inhibitor.

Compound **3** was then additionally docked using extra-precision Glide (Glide XP)^21^ and also redocked using QM-Polarized Ligand Docking (QPLD) protocol^22^ that generates partial charges on the ligand atoms by quantum mechanical calculations on the ligand in the field of the receptor. This accounts for the polarisation of the charges on the ligand by the receptor environment, and redocking with these new charges can lead to improved docking accuracy. The QPLD pose showed a difference of 2.54 Å from the original pose, mainly due to the *m*-tolyl ring flip (Figure 2(E)). Based on the putative binding mode, the carboxylic acid mimics the phosphate groups of ATP that coordinate Mg^2+^ ions (Figure 2(D,E)). As shown in Table 4, the electrostatic term (XP Electro) from Coulombic and metal interactions contributes the most, followed by XP Zpotr, which denotes a reward for ligand atoms placed in a favourable electrostatic environment of the protein, and XP PhobEn, a reward for hydrophobic enclosure. Thus, the appropriately positioned carboxylate group is mainly responsible for successful binding.

**Table 4.** The breakdown of GlideScore scores into individual contributions, only non-zero terms are shown.

	XP GScore	XP HBond	XP PhobEn	XP LowMW	XP LipophilicEvdW	XP Electro	XP Zpotr
Glide XP (original) pose	–12.549	–0.76	–2.00	–0.5	–1.664	–5.125	–2.5
QPLD pose	–12.009	–0.57	–2.05	–0.5	–1.261	–5.125	–2.5

To confirm the relevance of the predicted docking pose, a 100 ns molecular dynamics (MD) simulation on the **3** binding pose was run. The pose was fairly stable during the simulation time (Supplementary Figure S4), with the largest fluctuations resulting from the flipping of the *m*-tolyl ring. The metal coordinating and ionic interactions with both of the active site Mg^2+^ ions through the carboxylate moiety and one pyrimidino nitrogen anchored **3** in the active site (Supplementary Figure S5). Additionally, a cation-π interaction of the *m*-tolyl ring with Lys144 occurred quite frequently. Overall, in the absence of an experimental crystal structure, these results provide strong evidence for the proposed binding mode of compound **3** in the ATP-binding site.

The antibacterial activities of **3** were determined according to the European Committee for Antibacterial Susceptibility Testing recommendations and Clinical Laboratory Standards Institute protocol^23^. Compound was tested against reference strains of *E. coli* and *Staphylococcus aureus* and against two mutant *E. coli* strains. *E. coli* D22 bears a mutation in the LpxC gene that increases membrane permeability, whereas *E. coli* N43 is an AcrA knockout strain (knockout of the cell membrane pump). The **3** showed no antibacterial activity against both *E. coli* and *S. aureus* (Table S7). The most plausible reason is the low on target activity of the hit fragment, however, also the poor penetration into the bacterial cell or efflux pumps effect cannot be ruled out. Although the lack of antibacterial activity is a limitation, hit compound **3** represents a fragment-sized starting point for the development of new compounds with increased DdlB inhibitory potency as well as antibacterial activity.

## Conclusions

Here, we describe the construction of a fragment library containing 943 fragments to discover novel inhibitors of a bacterial enzyme DdlB involved in intracellular steps of peptidoglycan biosynthesis. FBDD is an innovative and underutilised approach to target DdlB. The library contained diverse fragments suitable for screening on different targets, as well as fragments specifically designed to target DdlB. Library screening using a biochemical assay was followed by profiling of the fragment hits. Compounds that interfered with the assay, redox active compounds, and reactive compounds were flagged. The fragment hit **3** without flags and with an IC_50_ value of 168 μM was selected for further studies. We confirmed binding of **3** to DdlB with SPR analysis. Furthermore, steady-state kinetics showed **3** to be a competitive inhibitor with respect to ATP with a Ki value of 20.7 ± 4.5 µM. An analogue-by-catalogue campaign did not reveal more potent compounds, making fragment hit **3** a starting point for further synthetic optimisation to develop new antibacterial agents.

## Experimental section

### Fragment library preparation

The *in silico* part of the fragment library preparation was performed using the KNIME analytics platform^24^. SMARTSview server was used to visualise the SMARTS patterns^25^. For fragment library preparation, we selected compounds from more than 4500 in-house chemicals and more than 1.57 million compounds available for cherry-picking from ChemDiv (the database was downloaded on 05 October 2020). First, the RDKit Descriptor Calculation node^26^ was used to calculate the molecular descriptors for each compound in the library. Then, the compounds were filtered using the following criteria to retain only fragments: molecular weight between 80 and 300, no more than 5 hydrogen bond donors, no more than 10 hydrogen bond acceptors, and estimated logP value no greater than 4. The Speedy SMILES Organic Subset Molecules Splitter node was used to remove compounds with non-organic atoms other than C, N, O, S, P, B, F, Cl, Br, I, and H. To remove reactive and unwanted functional groups, a set of SMARTS filters (Supplementary Table S1) were applied using the SMARTS Query node^27^. Finally, RDKit Molecule Catalogue Filter node was used to remove PAINS compounds known to cause interference in assay systems^28^. The resulting virtual fragment library of 1087 in-house and 91,379 commercially available compounds was then used to design six subsets.

The selected commercially available fragments were obtained at 1.0–1.2 mg on 96-well polypropylene plates. To each well, 100 μL of dimethyl sulfoxide (DMSO) was added, and the plates were covered with an aluminium seal, and sonicated to prepare 26–125 mM stock solutions, which were used for screening in a DdlB inhibition assay.

#### In-house diverse fragments

Additional physicochemical filters were applied to the in-house available fragments, namely no more than 5 hydrogen bond acceptors, no more than 3 rotatable bonds, and a polar surface area greater than 0 and no greater than 80, resulting in a library of 887 in-house available fragments. Then, 400 diverse fragments were selected using the RDKit Diversity Picker node. The node uses a fast MaxMin algorithm to select diverse compounds based on the Tanimoto distance between the Daylight-like topological fingerprints. The diverse fragments were then manually inspected and a set of 195 fragments was selected for the physical fragment library. The fragments were prepared as 50 mM DMSO stock solutions and transferred to 96-well polypropylene plates.

#### Purchased diverse fragments

From the 91,379 commercially available fragments, 2000 diverse fragments were selected using the RDKit Diversity Picker node. Subsequently, *k*-means clustering into 500 clusters was applied to the calculated distance matrix for Daylight-like topological fingerprints using the k-Medoids node. Next, we manually inspected 500 medoids and selected a set of 283 to be included in the physical fragment library.

#### Chelating fragments

The Brenk filter (Supplementary Table S1) without filters for phosphor and catechol was used in the previous step to prepare the subset of chelating fragments. Based on recent reviews^17^^,^^18^, we designed SMARTS patterns (Supplementary Table S2) to find fragments capable of metal ions chelation. The RDKit Substructure Filter was used in the KNIME analytics platform. When filtering with a particular SMARTS pattern resulted in more than 100 fragment chelators, the RDKit Diversity Picker node was used to select 100 diverse fragment chelators. A total of 214 fragments were then manually selected.

#### Phosphate bioisostere fragments

The Brenk filter (Supplementary Table S1) without filters for Michael acceptors was used in the previous step to prepare the subset of phosphate bioisosters. SMARTS patterns for phosphate bioisosteres were designed based on a review^19^. The RDKit Substructure Filter was used in the KNIME analytics platform. When filtering with a particular SMARTS pattern resulted in more than 100 phosphate bioisostere fragments, the RDKit Diversity Picker node was used to select 100 diverse fragment chelators. A total of 159 fragments were then manually selected.

#### Cycloserine analogues

We applied several SMARTS patterns (Supplementary Table S4) to find fragments containing similar substructures to cycloserine. The RDKit Substructure Filter was used in the KNIME analytics platform. A total of 31 cycloserine analogues were manually selected.

#### Docked fragments to DdlB

The final subset contained the highest scoring fragments from docking to DdlB. The DdlB protein was prepared from the X-ray structure in complex with ADP and D-cycloserine phosphate (PDB ID: 4C5A)^11^, using Protein Preparation Wizard (Schrödinger Suite 2020–2, Schrödinger, LLC, New York, NY, 2020)^29^. Briefly, hydrogen atoms were added, residues were protonated at pH 7.0, the hydrogen bonding network was refined, waters were removed, and restrained minimisation was performed. Next, Make Receptor 3.4.0.2 (OpenEye Scientific Software, Inc., Santa Fe, NM, USA; www.eyesopen.com) was used to define grid boxes for each of the binding sites separately. The ATP binding site was defined with a box of 4214 Å^3^ (14.39 Å × 14.37 Å × 20.38 Å) and the outer contour of 739 Å^3^. The D-Ala binding site where cycloserine binds was defined with a box of 2155 Å^3^ (12.71 Å × 11.43 Å × 14.84 Å) and the outer contour of 308 Å^3^. The virtual fragment library containing 42,700 commercially available fragments was first processed using FixpKa (OpenEye Scientific Software, Inc., Santa Fe, NM, USA; www.eyesopen.com) to obtain proper ionisation at pH 7.4. Subsequently, the stereoisomer and conformational model generator OMEGA 3.1.2.2 (OpenEye Scientific Software, Inc., Santa Fe, NM, USA; www.eyesopen.com) was used to enumerate stereocenters and to generate up to 200 conformers per compound. The prepared fragment library was then docked to each binding site individually using FRED 3.4.0.2 (OpenEye Scientific Software, Inc., Santa Fe, NM, USA; www.eyesopen.com). Validation of the docking protocol was performed by redocking the co-crystallised ADP and D-cycloserine phosphate yielding RMSD < 2.0 Å. The 200 highest scoring virtual screening hits for the ATP binding site were clustered into 50 clusters, and 36 diverse fragments were manually selected. The 400 highest scoring virtual screening hits for the ATP binding site were clustered into 60 clusters, and 25 diverse fragments were manually selected.

### Ddlb inhibition assay

A recombinant *E. coli* DdlB enzyme (D-alanine:D-alanine ligase) was expressed in *E. coli*^11^. Inhibition of the enzyme was determined using an end-point malachite green assay by detecting the orthophosphate formed during the enzymatic reaction. The final mixture (50 μL) contained: 50 mM HEPES, pH 8.0, 0.005% Triton X-114, 5 mM MgCl_2_, 6.5 mM (NH_4_)_2_SO_4_, 10 mM KCl, 700 μM D-Ala, 100 μM ATP, purified DdlB (diluted in 50 mM HEPES, pH 8.0), and the test compound dissolved in DMSO. The final DMSO concentration was 5%. After 20 min of incubation at 37 °C, the reaction was terminated by adding Biomol^®^ reagent (100 μL) and after 5 min at room temperature the absorbance was measured at 650 nm using a microplate reader (Synergy H4, BioTek Instruments, Inc., USA). A parallel experiment without the enzyme was performed to detect insoluble compounds under the assay conditions and subtracted from each measurement. All experiments were performed in duplicates. RAs were calculated with respect to blank experiments without tested compounds and with 5% DMSO. The final concentrations for fragment screening were 1.3–6.2 mM for purchased fragments and 2.5 mM for in-house available fragments. IC_50_ values were determined by measuring the residual activities in quadruplicate at seven different compound concentrations and calculated using in GraphPad Prism (GraphPad Software, San Diego, CA, USA).

For compound **3**, Ki values were determined against DdlB from *E. coli*. Ki determinations were performed under similar conditions as described for DdlB inhibition assay, where different concentration of one substrate and fixed concentration of the other were used. The concentration of ATP (50, 100, 200, 300 and 500 µM) was varied at fixed concentration of D-Ala (700 µM). The concentrations of compound **3** were 0, 25, 50, 100, 250, 500, and 1000 µM. After 20 min of incubation, 100 µM of Biomol^®^ green reagent was added, and absorbance was read at 650 nm after 5 min. Initial data were fitted to competitive, (*v*/*V* = [S]/{*K*_M_(1 + [I]/*K*_I_)+ [S]}), non-competitive (*v*/*V* = [S]/{(1 + [I]/*K*_I_)(*K*_M_+ [S])}), uncompetitive (*v*/*V* = [S]/{*K*_M_+ [S](1 + [I]/*K*_I_)}) inhibition models in which *v* is observed reaction rate, *V* is the maximum rate, *K*_M_ is the Michaelis constant, using SigmaPlot 12.0 software. The best fit was taken as the one having the highest *R*^2^, the lowest standard error and the narrowest 95% confidence interval in each parameter (see Supplementary Table S6).

### Mura inhibition assay

A recombinant *E. coli* MurA enzyme (UDP-*N*-acetylglucosamine enolpyruvyl transferase) was expressed in *E. coli*^30^. Inhibition of the enzyme was determined using an end-point malachite green assay by detecting the orthophosphate formed during the enzymatic reaction. The final mixture (50 μL) contained: 50 mM HEPES, pH 8.0, 0.005% Triton X-114, 200 μM UDP-*N*-acetylglucosamine, 100 μM phosphoenolpyruvate, purified MurA (diluted in 50 mM HEPES, pH 8.0), and 5 mM of each test compound dissolved in DMSO. The final DMSO concentration was 5%. After 15 min of incubation at 37 °C, the reaction was terminated by adding Biomol^®^ reagent (100 μL), and after 5 min at room temperature the absorbance was measured at 650 nm using a microplate reader (Synergy H4, BioTek Instruments, Inc., USA). A parallel experiment without the enzyme was performed to detect insoluble compounds under the assay conditions and subtracted from each measurement. All experiments were performed in duplicates. RAs were calculated with respect to blank experiments without tested compounds and with 5% DMSO.

### Phosphate-binding assay

Phosphate binding was determined by colorimetric measurement of phosphate with malachite green in the presence or absence of the compound. To 46.9 μL of buffer (50 mM HEPES, pH 8.0), 0.625 μL of 800 μM phosphate standard, and 2.5 μL of 100 mM compound DMSO stock was added. The reaction mixture was incubated for 5 min at room temperature. Then, 100 μL of Biomol^®^ reagent was added and after 5 min, the absorbance was measured at 650 nm using a microplate reader (Synergy H4, BioTek Instruments, Inc., USA). Background absorbance was determined in a parallel experiment without phosphate standard and subtracted from each measurement. To determine the blank value, the compound solution was replaced with pure DMSO and the background-subtracted measurements were divided by the blank value.

### Redox activity assays

#### HRP-PR assay

The HRP-PR assay was performed according to a previously optimised procedure^31^. Briefly, to 58 μL of buffer (50 mM HEPES, 50 mM NaCl, pH 7.5), 10 μL of 2 mM compound DMSO stock solution, and 66 μL of buffer (redox-free) or 66 μL of 3 mM DTT in buffer were added. After 15 min of incubation at room temperature, 66 μL of HRP-PR detection reagent (300 µg/mL phenol red and 180 µg/mL HRP [150–250 units/mg solid] in buffer) was added and incubated for an additional 5 min at room temperature. The final concentrations were: 100 μM compound, 100 μg/mL (282 μM) phenol red, 60 μg/mL HRP, 1 mM DTT, 5% DMSO. The reaction was then quenched by adding 10 μL of 1 M NaOH solution and the absorbance was measured at 610 nm using a microplate reader (Synergy H4, BioTek Instruments, Inc., USA). To determine the blank value, the compound solution was replaced with pure DMSO. The measured absorbance for each compound was then divided by the blank value. 3-Methyltoxoflavin was used as a control compound. All reagent solutions were freshly prepared before performing the experiments.

#### H_2_DCFDA assay

The H_2_DCFDA assay was performed according to a previously optimised procedure^31^. Briefly, the probe H_2_DCFDA was prepared as a 5 mM stock in DMSO and diluted 10-fold to 500 µM with 0.01 M NaOH. The probe was freshly prepared for each plate and stored in the dark at room temperature for 30 min to hydrolyse the ester. To 52.5 μL of buffer (50 mM HEPES, 50 mM NaCl, pH 7.5), 7.5 μL of 2 mM compound DMSO stock solution, 75 μL of buffer (redox-free) or 75 μL of 200 μM TCEP in buffer were added, and 15 μL of 500 µM H_2_DCFDA. The final concentrations were: 100 μM compound, 50 μM H_2_DCFDA, 100 μM TCEP, and 5% of DMSO. The microplate was covered with a lid and stored in the dark at room temperature for 30 min. Fluorescence intensity was then measured using 485 nm excitation and 535 nm emission filters (Synergy H4, BioTek Instruments, Inc., USA). To determine the blank value, the compound solution was replaced with pure DMSO. The measured fluorescence for each compound was then divided by the blank value. 3-Methyltoxoflavin was used as a control compound. All reagent solutions were freshly prepared before performing the experiments.

#### Resazurin assay

The resazurin assay was performed according to a previously optimised procedure^31^. Briefly, to 100 μL of buffer (50 mM HEPES, 50 mM NaCl, pH 7.5), 2 μL of 1, 0.1, or 0.01 mM compound DMSO stock solution, and 100 μL of resazurin solution (10 μM resazurin and 200 μM DTT in buffer) were added. The final concentrations were: 0.1, 1, or 10 μM compound, 5 μM resazurin, 100 μM DTT, and 1% of DMSO. The microplate was covered with a lid and stored in the dark at room temperature for 30 min. Fluorescence intensity was then measured using 560 nm excitation and 590 nm emission filters (Synergy H4, BioTek Instruments, Inc., USA). To determine the blank value, the compound solution was replaced with pure DMSO. The measured fluorescence for each compound was then divided by the blank value. 3-Methyltoxoflavin was used as a control compound. All reagent solutions were freshly prepared before performing the experiments.

### Thiol reactivity assay

The assay was performed as previously described^31^. Briefly, 100 μM of compound was incubated at 37 °C with a mixture of 100 μM TCEP and 25 μM DTNB (generating 50 μM TNB^2−^ *in situ*) in buffer (20 mM sodium phosphate, 150 mM NaCl, pH 7.4) with 5% final DMSO concentration. To monitor TNB^2−^ depletion, absorbance at 412 nm was measured every 5 min for 12 h (Synergy H4, BioTek Instruments, Inc., USA). Compound background absorbance was subtracted from each measurement. 2-Chloro-*N*-(3-chlorophenyl)acetamide was used as a control compound. All reagent solutions were freshly prepared before performing the experiments.

### Surface plasmon resonance

The SPR measurements were performed at the Infrastructural Centre for Analysis of Molecular Interactions at the Department of Biology, University of Ljubljana on a Biacore T200 (GE Healthcare) at 25 °C. To assay the interaction of **3** compound with the DdlB protein we immobilised approximately 7700 RU of the purified DdlB protein on the carboxymethyl dextran-coated gold surface of the series S sensor chip CM5 (GE Healthcare) to which DdlB was immobilised *via* the free amino groups by an amine coupling procedure^32^. For this, DdlB protein was in 10 mM sodium acetate buffer (pH 4). Compound **3** was serially diluted in running buffer composed of 50 mM HEPES (pH 7.4), 140 mM NaCl, 0.005% P20. To assay the fragment**–**DdlB interaction, each fragment concentration tested (ranging from 78.125 to 5000 µM) for 40 s at a flow rate of 30 μL/min and dissociation was followed for 40 s. For the regeneration of the surface of the sensor chip, 2 mM NaOH was used. As the stock of the compound was prepared in 5% DMSO, we referenced the sensorgrams for the buffer response containing identical concentration of DMSO as the diluted sample of the compound. Furthermore, sensorgrams were referenced for the untreated surface flow-cell 1 response. The data were analysed with the Biacore T200 Evaluation Software and *K_D_* was determined by fitting the data to the steady state affinity model. The average *K_D_*s and standard deviations were determined from three titrations of **3**.

### Microbiological evaluation

Minimum inhibitory concentrations (MICs) for **3** were determined by broth microdilution in Cation-adjusted Mueller-Hinton broth (MH) with TES, TREK Diagnostic Systems Ltd. against *S. aureus* ATCC 29213, *E. coli* ATCC 25922, *E. coli* N43 and *E. coli* D22. All of the experiments were performed in duplicate in 96-well plate format according to CLSI guidelines and European Committee for Antimicrobial Susceptibility Testing recommendations. The bacterial suspension of a specific bacterial strain was diluted with MH broth to obtain a final inoculum of 5 × 10^5^ CFU/mL in the assay. Compound **3** dissolved in DMSO and inoculum were mixed together and incubated for 20 h at 35 °C. After incubation, the minimal inhibitory concentration (MIC) values were determined by visual inspection as the lowest dilution of compounds showing no turbidity. The MICs were determined against *S. aureus* (ATCC 29213), *E. coli* (ATCC 25922), *E. coli* N43, and *E. coli* D22 bacterial strains. D-Cycloserine was used as a positive control.

### High-precision docking and molecular dynamics

The DdlB protein was prepared from the X-ray structure in complex with ADP and D-Ala-D-Ala (PDB ID: 4C5C) using Protein Preparation Wizard^29^ (Schrödinger Suite 2021–1, Schrödinger, LLC, New York, NY, 2021). Briefly, hydrogen atoms were added, residues were protonated at pH 7.0, the hydrogen bonding network was refined, waters were removed, and restrained minimisation was performed. Only chain A was retained. The receptor grid was generated using Receptor Grid Generation with van der Waals radii scaling by 1, partial charge cut-off 0.25 (default settings), and OPLS_2005 forcefield^33^, while the co-crystallised ADP defined the active site with the inner box of 10 Å^3^ and outer box of 23.2 Å^3^. The structure of **3** was prepared with LigPrep, ionised with Epik^34^ (pH 7 ± 2), and additional metal binding states were added. Docking was performed using Glide XP^21^ with the default settings. The top output pose was visualised in Maestro, and then redocked using QM-Polarized Ligand Docking protocol^22^ with default settings, accurate QM treatment (B3LYP/LACVP*), and XP precision.

For molecular dynamics, the docking pose of **3** was merged with the crystal structure, then the system was prepared with System Builder: TIP3P^35^ water molecules were added up to 10 Å from the protein surface to solvate the protein in an orthorhombic box, K^+^ and Cl^–^ ions were added to neutralise the system and produce the final 0.15 M^36^ concentration, and OPLS_2005 force field^33^ was used for parametrisation of the macromolecule as well as the ligand. Desmond^37^ was used for molecular dynamics. The default Desmond relaxation protocol (desmond_npt_relax.msj) was used for the equilibration stage of DdlB structure: (1) 100 ps of Brownian Dynamics NVT, 10 K, small timesteps, with restraints on the solute heavy atoms, (2) 12 ps NVT, 10 K, with small timesteps and restraints on the solute heavy atoms, (3) 12 ps NPT, 10 K, and restraints on the solute heavy atoms, and (4) 24 ps unrestrained NPT; followed by the production stage: 1.2 ps interval for energy, RESPA integrator with 2 fs time step, cut-off scheme at 9.0 Å, random seed, isothermal-isobaric NPT ensemble at 300 K and 1.013 bar pressure with Nose-Hoover chain thermostat and Martyna-Tobias-Klein barostat (1 and 2 ps relaxation time, respectively, with isotropic coupling). The simulation time was 100 ns with 1000 frames per trajectory. The simulation results were analysed using the built-in Desmond tools.

### Compound characterisation

Compound were purchased from several vendors (ChemBridge Corporation, ChemDiv, Enamine, Maybridge, Vitas-M Laboratory, and Wuxi LabNetwork) and used as received. The identity of compounds was confirmed with nuclear magnetic resonance, recorded on a Bruker Avance III 400 MHz spectrometer, using DMSO-*d*_6_ as a solvent. Chemical shifts are reported in *parts per million* (ppm), and the central peak of the residual solvent resonance at 2.50 ppm was used as the reference. The multiplicities are reported as follows: *s* (singlet), *d* (doublet), *t* (triplet), *q* (quartet), *m* (multiplet), *dd* (doublet of doublets), *ddd* (doublet doublet of doublets), *tt* (triplet of triplets), *dt* (doublet of triplets), *td* (triplet of doublets), and *qi* (quartet of doublets), coupling constants (*J*) quoted in Hertz (Hz), number of equivalent nuclei (by integration). To further confirm the identity of compounds, high-resolution mass spectrometry measurements were performed on a Thermo Scientific Q Exactive Plus Hybrid Quadrupole-Orbitrap mass spectrometer (Thermo Fisher Scientific, Waltham, MA, USA). Compound purity was determined by HPLC analysis on Thermo Scientific Dionex UltiMate 3000 modular system (Thermo Fisher Scientific Inc.) with Waters Acquity UPLC^®^ HSS C18 SB column (2.1 × 50 mm, 1.8 µm) thermostated at 40 °C, injection volume, 1 µL; flow rate, 0.3 mL/min; detector *λ*, 220 nm and 254 nm; mobile phase A: 0.1% TFA (v/v) in water; mobile phase B: MeCN. Method: 0–9 min, 5–95% B; 9–11 min, 95% B; 11–11.5 min, 95–5% B. Compounds are >95% pure by HPLC analysis, unless stated otherwise.

#### 7-(m-Tolyl)pyrazolo[1,5-a]pyrimidine-3-carboxylic acid (3, Vitas-M laboratory, STL396729)


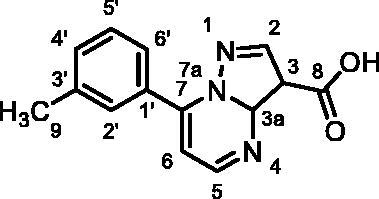


^1^H NMR (400 MHz, DMSO-*d*_6_) δ 13.36 (s, 1H, COOH), 8.70 (d, *J* = 4.3 Hz, 1H, C5-H), 7.92 (dt, *J* = 7.7, 1.7 Hz, 1H, C6’-H), 7.89 − 7.84 (m, 1H, C2'-H), 7.52 (t, *J* = 7.6 Hz, 1H, C5’-H), 7.49 − 7.43 (m, 1H, C4’-H), 7.35 (d, *J* = 4.3 Hz, 1H, C6-H), 7.21 (s, 1H, C2-H), 2.43 (s, 3H, CH_3_). ^13^C NMR (101 MHz, DMSO-*d*_6_) δ 163.95 (COOH), 151.16 (C5), 150.09 (C3), 148.02 (C1'), 146.70 (C7), 138.39 (C3'), 132.26 (C4'), 130.67 (C3a), 130.23 (C2'), 128.93 (C5'), 127.12 (C6'), 110.15 (C6), 99.38 (C2), 21.50 (CH_3_). HRMS (ESI^+^) *m/z* [M + H]^+^, calcd. for C_14_H_12_N_3_O_2_: 254.09240, found: 254.09159. Purity by HPLC: 95.6%. 2 D NMR spectra are shown in Supplementary Figures S6–S12.

#### (2-Ethoxy-7,8-dihydro-1,6-naphthyridin-6(5H)-yl)(pyridin-2-yl)methanone (6, ChemDiv, S422-0111)


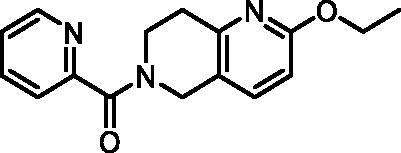


^1^H NMR (400 MHz, DMSO-*d*_6_) δ 8.63 (ddd, *J* = 8.1, 4.4, 2.6 Hz, 1H), 8.01 − 7.90 (m, 1H), 7.68 − 7.58 (m, 2H), 7.57 − 7.47 (m, 1H), 6.67 (d, *J* = 8.4 Hz, 1H), 4.74 (s, 1H), 4.57 (s, 1H), 4.26 (q, *J* = 7.0 Hz, 2H), 3.96 (t, *J* = 6.0 Hz, 1H), 3.68 (t, *J* = 5.9 Hz, 1H), 2.97 − 2.80 (m, 2H), 1.30 (td, *J* = 7.0, 2.0 Hz, 3H). Purity by HPLC: 92.6%.

#### 6-Amino-4-hydroxyquinoline-3-carboxylic acid (7, ChemBridge, 9220711)


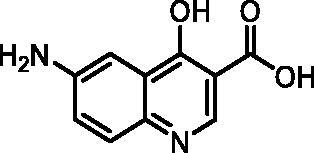


^1^H NMR (400 MHz, DMSO-*d*_6_) δ 8.61 (s, 1H), 7.55 (d, *J* = 8.9 Hz, 1H), 7.31 (d, *J* = 2.6 Hz, 1H), 7.18 (dd, *J* = 8.9, 2.6 Hz, 1H), 5.81 (s, 2H). Purity by HPLC: 98.5%.

#### 7-Phenylpyrazolo[1,5-a]pyrimidine-3-carboxylic acid (9, Enamine, EN300-39113)


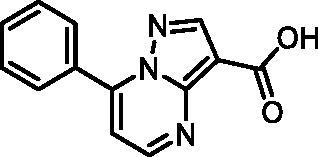


^1^H NMR (400 MHz, DMSO-*d*_6_) δ 12.46 (s, 1H), 8.86 (d, *J* = 4.5 Hz, 1H), 8.63 (s, 1H), 8.15 − 8.06 (m, 2H), 7.70 − 7.57 (m, 3H), 7.46 (d, *J* = 4.4 Hz, 1H). Purity by HPLC: 98.8%.

#### 7-(p-Tolyl)pyrazolo[1,5-a]pyrimidine-3-carboxylic acid (10, ChemBridge, 8928448)


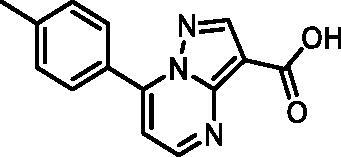


^1^H NMR (400 MHz, DMSO-*d*_6_) δ 8.82 (d, *J* = 4.5 Hz, 1H), 8.62 (s, 1H), 8.06 − 7.99 (m, 2H), 7.47 − 7.40 (m, 3H), 2.43 (s, 3H). Purity by HPLC: 98.0%.

#### 7–(3,4-Dimethylphenyl)pyrazolo[1,5-a]pyrimidine-3-carboxylic acid (11, Vitas-M laboratory, STK902058)


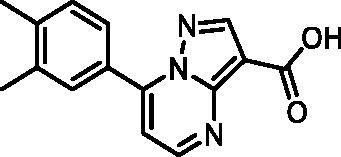


^1^H NMR (400 MHz, DMSO-*d*_6_) δ 12.40 (s, 1H), 8.81 (d, *J* = 4.5 Hz, 1H), 8.63 (s, 1H), 7.92 − 7.82 (m, 2H), 7.42 (d, *J* = 4.5 Hz, 1H), 7.38 (d, *J* = 8.0 Hz, 1H), 2.34 (s, 3H), 2.33 (s, 3H). Purity by HPLC: 93.0%.

#### 7–(4-Chlorophenyl)pyrazolo[1,5-a]pyrimidine-3-carboxylic acid (12, ChemDiv, C201-1701)


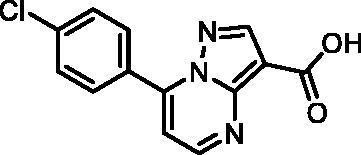


^1^H NMR (400 MHz, DMSO-*d*_6_) δ 12.46 (s, 1H), 8.86 (s, 1H), 8.63 (d, *J* = 3.9 Hz, 1H), 8.17 − 8.10 (m, 2H), 7.74 − 7.67 (m, 2H), 7.49 (s, 1H). Purity by HPLC: 98.6%.

#### 7–(3-Nitrophenyl)pyrazolo[1,5-a]pyrimidine-3-carboxylic acid (13, Vitas-M laboratory, STK349882)


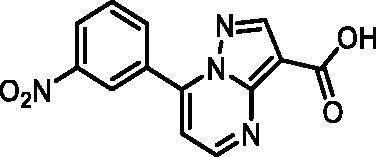


^1^H NMR (400 MHz, DMSO-*d*_6_) δ 12.50 (s, 1H), 8.98 (t, *J* = 2.0 Hz, 1H), 8.91 (d, *J* = 4.4 Hz, 1H), 8.67 (s, 1H), 8.53 − 8.45 (m, 2H), 7.93 (t, *J* = 8.1 Hz, 1H), 7.62 (d, *J* = 4.4 Hz, 1H). Purity by HPLC: 98.1%.

#### 7-(Benzo[d][1,3]dioxol-5-yl)pyrazolo[1,5-a]pyrimidine-3-carboxylic acid (14, Vitas-M laboratory, STK902056)


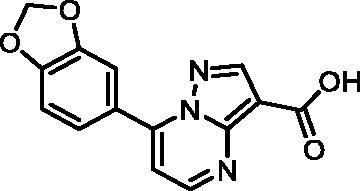


^1^H NMR (400 MHz, DMSO-*d*_6_) δ 8.76 (d, *J* = 4.5 Hz, 1H), 8.56 (s, 1H), 7.75 − 7.68 (m, 2H), 7.38 (d, *J* = 4.5 Hz, 1H), 7.19 − 7.13 (m, 1H), 6.17 (s, 2H). Purity by HPLC: 98.2%.

#### 7-(Thiophen-2-yl)pyrazolo[1,5-a]pyrimidine-3-carboxylic acid (15, Vitas-M laboratory, BBL039910)


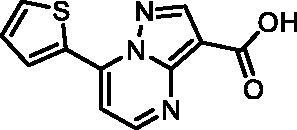


^1^H NMR (400 MHz, DMSO-*d*_6_) δ 13.51 (s, 1H), 8.68 (d, *J* = 4.6 Hz, 1H), 8.58 (dd, *J* = 3.9, 1.2 Hz, 1H), 8.14 (dd, *J* = 5.1, 1.2 Hz, 1H), 7.87 (d, *J* = 4.7 Hz, 1H), 7.41 (dd, *J* = 5.1, 3.9 Hz, 1H), 7.20 (s, 1H). HRMS (ESI^+^) *m/z* [M + H]^+^, calcd. for C_11_H_8_N_3_O_2_S: 246.03317, found: 246.03262. Purity by HPLC: 99.3%.

#### 7-(Thiophen-3-yl)pyrazolo[1,5-a]pyrimidine-3-carboxylic acid (16, Wuxi LabNetwork, WXCD00314351)


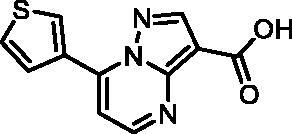


^1^H NMR (400 MHz, DMSO-*d*_6_) δ 12.43 (s, 1H), 9.16 (dd, *J* = 3.0, 1.3 Hz, 1H), 8.81 (d, *J* = 4.6 Hz, 1H), 8.70 (s, 1H), 8.08 (dd, *J* = 5.2, 1.4 Hz, 1H), 7.82 (dd, *J* = 5.2, 3.0 Hz, 1H), 7.76 (d, *J* = 4.7 Hz, 1H). Purity by HPLC: 98.9%.

#### 7–(1,3-Dimethyl-1H-pyrazol-4-yl)pyrazolo[1,5-a]pyrimidine-3-carboxylic acid (17, ChemBridge, 8909427)


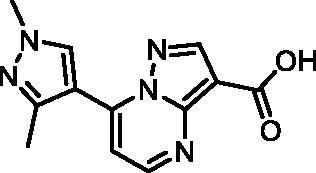


Purity by HPLC: 95.7%.

#### 7-(Thiophen-2-yl)pyrazolo[1,5-a]pyrimidine-2-carboxylic acid (18, Vitas-M laboratory, STK349529)


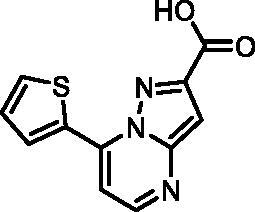


^1^H NMR (400 MHz, DMSO-*d*_6_) δ 13.45 (s, 1H), 8.69 (d, *J* = 4.7 Hz, 1H), 8.58 (dd, *J* = 3.9, 1.2 Hz, 1H), 8.14 (dd, *J* = 5.0, 1.2 Hz, 1H), 7.88 (d, *J* = 4.7 Hz, 1H), 7.41 (dd, *J* = 5.1, 3.9 Hz, 1H), 7.23 (s, 1H). Purity by HPLC: 96.6%.

#### 7-Phenylpyrazolo[1,5-a]pyrimidine-2-carboxylic acid (19, Vitas-M laboratory, STK350440)


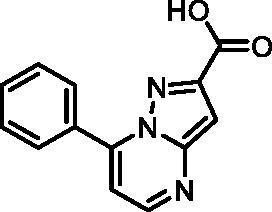


^1^H NMR (400 MHz, DMSO-*d*_6_) δ 13.37 (s, 1H), 8.71 (d, *J* = 4.3 Hz, 1H), 8.15 − 8.05 (m, 2H), 7.70 − 7.59 (m, 3H), 7.38 (d, *J* = 4.3 Hz, 1H), 7.22 (s, 1H). Purity by HPLC: 96.9%.

#### Ethyl 7–(3,4-dimethylphenyl)pyrazolo[1,5-a]pyrimidine-3-carboxylate (20, Vitas-M laboratory, STK902137)


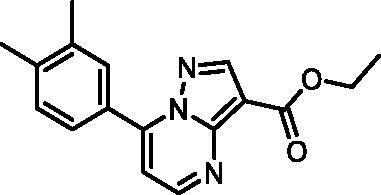


^1^H NMR (400 MHz, DMSO-*d*_6_) δ 8.85 (d, *J* = 4.5 Hz, 1H), 8.67 (s, 1H), 7.91 − 7.82 (m, 2H), 7.45 (d, *J* = 4.5 Hz, 1H), 7.38 (d, *J* = 8.0 Hz, 1H), 4.32 (q, *J* = 7.1 Hz, 2H), 2.34 (s, 3H), 2.33 (s, 3H), 1.33 (t, *J* = 7.1 Hz, 3H). Purity by HPLC: 98.4%.

#### 7-Phenylpyrazolo[1,5-a]pyrimidine-3-carboxamide (21, Vitas-M laboratory, STK649786)


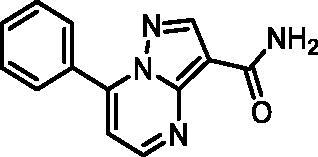


^1^H NMR (400 MHz, DMSO-*d*_6_) δ 8.85 (d, *J* = 4.5 Hz, 1H), 8.60 (s, 1H), 8.17 − 8.09 (m, 2H), 7.71 − 7.58 (m, 4H), 7.55 (s, 1H), 7.47 (d, *J* = 4.5 Hz, 1H). Purity by HPLC: 99.0%.

#### 7-(m-Tolyl)pyrazolo[1,5-a]pyrimidine-3-carbonitrile (22, ChemBridge, 8909699)


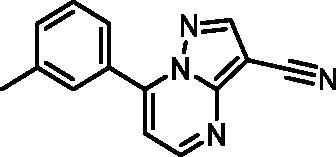


^1^H NMR (400 MHz, DMSO-*d*_6_) δ 8.92 − 8.84 (m, 2H), 7.93 − 7.85 (m, 2H), 7.59 − 7.44 (m, 3H), 2.42 (s, 3H). Purity by HPLC: 94.0%.

#### (Z)-N'-Hydroxy-7–(3-(trifluoromethyl)phenyl)pyrazolo[1,5-a]pyrimidine-3-carboximidamide (23, Maybridge, SEW04848)


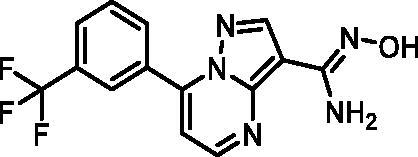


^1^H NMR (400 MHz, DMSO-*d*_6_) δ 9.43 (d, *J* = 1.1 Hz, 1H), 8.72 (dd, *J* = 4.4, 1.1 Hz, 1H), 8.50 (s, 1H), 8.45 (d, *J* = 1.1 Hz, 1H), 8.38 (d, *J* = 8.1 Hz, 1H), 8.01 (d, *J* = 7.9 Hz, 1H), 7.87 (t, *J* = 7.9 Hz, 1H), 7.43 (dd, *J* = 4.4, 1.1 Hz, 1H), 6.03 (s, 2H). Purity by HPLC: 98.5%.

## Supplementary Material

Supplemental Material
