# Supplementary material for: Discovery of a fragment hit compound targeting D-Ala:D-Ala ligase of bacterial peptidoglycan biosynthesis
Source: J Enzyme Inhib Med Chem. 2022 Nov 29;38(1):387–97. doi: 10.1080/14756366.2022.2149745 (PMC9718554; doi:10.1080/14756366.2022.2149745)
Supplement: Supplemental Material [file IENZ_A_2149745_SM2502.zip › IENZ_2149745_SuppMat.pdf]

Supplemental material

Discovery of a fragment hit compound targeting D-Ala:D-Ala ligase of bacterial peptidoglycan biosynthesis

Matic Proj [a], Martina Hrast [a], Gregor Bajc [b], Rok Frlan [a], Anže Meden [a], Matej Butala [b], Stanislav Gobec [a,*]

[a] *University of Ljubljana, Faculty of Pharmacy, Department of Pharmaceutical Chemistry, Slovenia*

[b] *University of Ljubljana, Biotechnical Faculty, Department of Biology, Slovenia*

* *Corresponding author. E-mail address: stanislav.gobec@ffa.uni-lj.si (S. Gobec)*

Table of contents

1 Supplemental tables S2

2 Supplemental figures S11

3 HPLC traces S15

4 NMR characterization S21

5 Supplemental references S27

Information Classification: General

1 Supplemental tables

Table S1. SMARTS patterns used to remove reactive and unwanted functional groups from compound libraries, based on Brenk filters [1].

Name	SMARTS pattern
Acid halide	C(=O)[Cl,Br,I,F]
Acyl cyanide	N#CC(=O)
Aldehyde	[CH1](=O)
Aliphatic long chain	[R0;D2][R0;D2][R0;D2][R0;D2]
Alkyl halide	[CX4][Cl,Br,I]
Azido group	N=[N+]=[N-]
Azo group	N#N
Azocane	[CH2R2]1N[CH2R2][CH2R2][CH2R2][CH2R2][CH2R2][CH2R2]1
Benzidine	[cR2]1[cR2][cR2]([Nv3X3,Nv4X4])[cR2][cR2][cR2]1[cR2]2[cR2][cR2][cR2]([Nv3X3,Nv4X4])[cR2][cR2]2
Betaketo/anhydride	[C,c](=O)[CX4,CR0X3,O][C,c](=O)
Biotin analogue	C12C(NC(N1)=O)CSC2
Carbo cation/anion	[C+,c+,C-,c-]
Catechol	c1c([OH])c([OH,NH2,NH])ccc1
Charged oxygen or sulfur atoms	[O+,o+,S+,s+]
Chinone	C1(=[O,N])C=CC(=[O,N])C=C1
Chinone_2	C1(=[O,N])C(=[O,N])C=CC=C1
Conjugated nitrile group	C=[C!r]C#N
Crown ether	[OR2,NR2]@[CR2]@[CR2]@[OR2,NR2]@[CR2]@[CR2]@[OR2,NR2]
Cumarine	c1ccc2c(c1)ccc(=O)o2
Cyanamide	N[CH2]C#N
Cyanate/aminonitrile/thiocyanate	[N,O,S]C#N
Cyanohydrins	N#CC[OH]
Diaminobenzene	[cR2]1[cR2]c([N+0X3R0,nX3R0])c([N+0X3R0,nX3R0])[cR2][cR2]1
Diaminobenzene_2	[cR2]1[cR2]c([N+0X3R0,nX3R0])[cR2]c([N+0X3R0,nX3R0])[cR2]1
Diaminobenzene_3	[cR2]1[cR2]c([N+0X3R0,nX3R0])[cR2][cR2]c1([N+0X3R0,nX3R0])
Diazo group	[N!R]=[N!R]
Diketo group	[C,c](=O)[C,c](=O)
Disulphide	SS
Ester of HOBT	C(=O)Onnn
Four member lactones	C1(=O)OCC1

Information Classification: General

Heavy metal	[Hg,Fe,As,Sb,Zn,Se,se,Te,Si]
Het-C-het not in ring	[NX3R0,NX4R0,OR0,SX2R0][CX4][NX3R0,NX4R0,OR0,SX2R0]
Hydrazine	N[NH2]
Hydroquinone	[OH]c1ccc([OH,NH2,NH])cc1
Iodine	I
Isocyanate	N=C=O
Ketene	C=C=O
Methylidene-1,3-dithiole	S1C=CSC1=S
Michael acceptor	C=!@CC=[O,S]
Michael acceptor_2	[$([CH]),$(CC)]#CC(=O)[C,c]
Michael acceptor_3	[$([CH]),$(CC)]#CS(=O)(=O)[C,c]
Michael acceptor_4	C=C(C=O)C=O
Michael acceptor_5	[$([CH]),$(CC)]#CC(=O)O[C,c]
N oxide	[NX2,nX3][OX1]
N-acyl-2-amino-5-mercapto-1,3,4-thiadiazole	s1c(S)nnc1NC=O
N-C-halo	NC[F,Cl,Br,I]
N-halo	[NX3,NX4][F,Cl,Br,I]
N-hydroxyl pyridine	n[OH]
N-nitroso	[#7]-N=O
Perfluorinated chain	[CX4](F)(F)[CX4](F)F
Peroxide	OO
Phenol ester	c1ccccc1OC(=O)[#6]
Phenyl carbonate	c1ccccc1OC(=O)O
Phosphor	P
Polyene	[CR0]=[CR0][CR0]=[CR0]
Quaternary nitrogen	[s,S,c,C,n,N,o,O]~[nX3+,NX3+](~[s,S,c,C,n,N])~[s,S,c,C,n,N]
Quaternary nitrogen_2	[s,S,c,C,n,N,o,O]~[n+,N+](~[s,S,c,C,n,N,o,O])(~[s,S,c,C,n,N,o,O])~[s,S,c,C,n,N,o,O]
Quaternary nitrogen_3	[*]=[N+]=[*]
Saponine derivative	O1CCCCC1OC2CCC3CCCCC3C2
Silicon halogen	[Si][F,Cl,Br,I]
Stilbene	c1ccccc1C=Cc2ccccc2
Sulfinic acid	[SX3](=O)[O-,OH]
Sulfonic acid_2	[C,c]S(=O)(=O)O[C,c]
Sulfonic acid_3	S(=O)(=O)[O-,OH]
Sulfonyl cyanide	S(=O)(=O)C#N

Information Classification: General

Sulfur oxygen single bond	[SX2]O
Sulphate	OS(=O)(=O)[O-]
Sulphur nitrogen single bond	[SX2H0][N]
Thiobenzothiazole	c12ccccc1(SC(S)=N2)
Thiobenzothiazole_2	c12ccccc1(SC(=S)N2)
Thiocarbonyl group	[C,c]=S
Thioester	SC=O
Thiol	[S-]
Thiol_2	[SH]
Three-membered heterocycle	*1[O,S,N]*1
Triflate	OS(=O)(=O)C(F)(F)F
Triphenyl methylsilyl	[SiR0,CR0](c1ccccc1)(c2ccccc2)(c3ccccc3)

Table S2. SMARTS patterns used in the preparation of chelating fragments library subset.

**Name**	**SMARTS pattern**	**Representation**	**Fragments purchased**
Hydroxamic acid	C(=O)N([H])O[H]	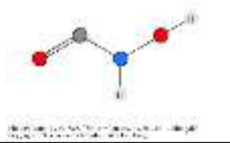	1
α-Hydroxy-β-aminoamide	NC(=O)C(O[H])CN	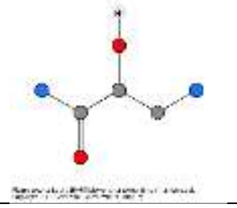	1
Quinolin-8-ol	[H]O(c1c2naaaa2aaa1)	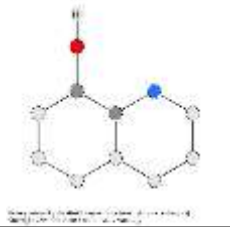	21

Information Classification: General

1,2,3-Triazole	[cR1]1[nR1]([H])[nR1][nR1][cR1]1	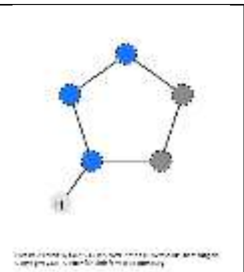	0
	[cR1]1[nR1][nR1]([H])[nR1][cR1]1	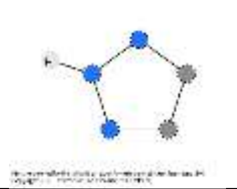	
1,2,4-Triazole	[cR1]1[nR1][cR1][nR1][nR1]([H])1	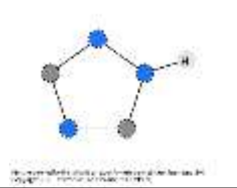	30
	[cR1]1[nR1]([H])[cR1][nR1][nR1]1	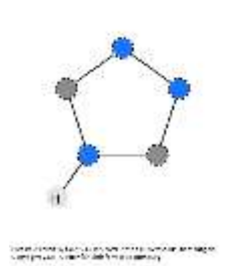	
Picolinic acid analogues	ncC(=O)O[H]	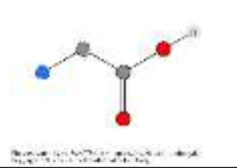	67
Pyrrolopyridine	c1n([H])c2ncaca2a1	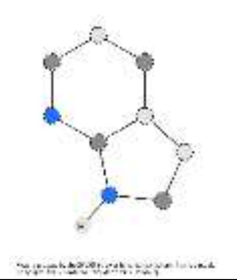	12
Quinolin-8-carboxylic acid	O=C(c1c2naaaa2aaa1)O[H]	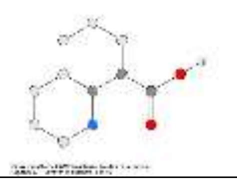	3

Information Classification: General

Pyrimidin-2-ol	n1c(O[H])naaa1	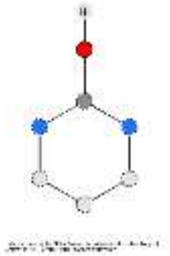	11
Hydroxypyridinones	[O,S]=c1cc[o,n]cc1O[H]	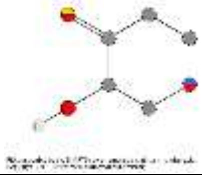	1
Salicylates	[H][O,N]c1c(C(O[H])=O)aaaa1	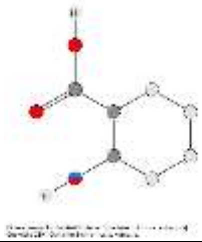	43
β,γ-Dihydroxy ketone	c(O[H])c(O[H])c[C,c]=O	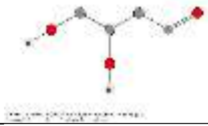	1
β-Ketocarboxylic acid	[C,c](=O)[C,c]C(=O)O[H]	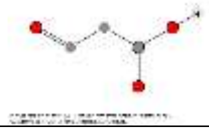	13
Hydroxymethyl ketone	C(=O)CO[H]	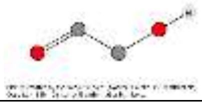	10

Information Classification: General

Table S3. SMARTS patterns used in the preparation of phosphate bioisostere fragments library subset.

**Name**	**SMARTS pattern**	**Representation**	**Fragments purchased**
Thiophosphonate	P([O;H,-])(O)=S	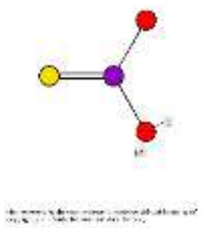	0
Carboxylate	C(=O)[O;H,-]	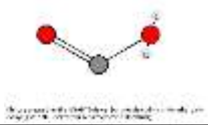	44
Benzoxazole-2-one	[N,n]([H])1[C,c](=O)[O,o]cc1	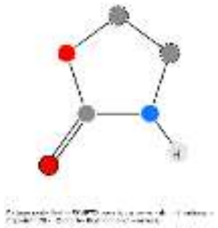	9
Sulfonate	[C,c]S(=O)(=O)[O;H,-]	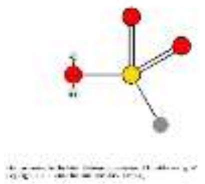	23
Sulfonamide	S(=O)(=O)N([H])([H])	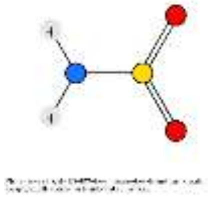	30
Squaric acid	C(=O)1C=C(O)C(=O)1	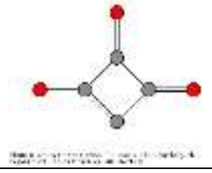	14

Information Classification: General

Squaramide	C(=O)1C=C(N)C(=O)1	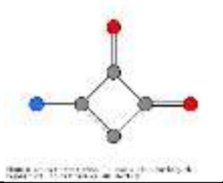	18
Boronic acid	B([O;H,-])(O)	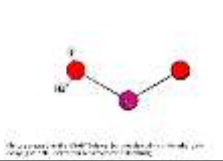	12
Thiazolidinone	C1C(=O)NCS1	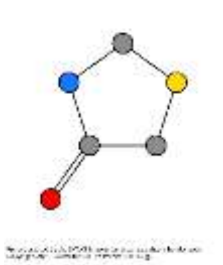	8
Tetronic acid	O=C1OCC(O[H])=C1	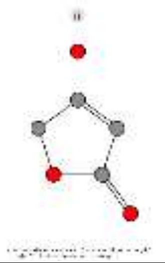	1

Information Classification: General

Table S4. SMARTS patterns used to select cycloserine analogues.

Name	SMARTS pattern	Representation	Fragments purchased
Isoxazol-3-ol	[C,c]1[C,c](O([H]))[N,n][O,o][C,c]1	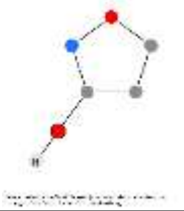	0
Isoxazol-3-one	[C,c]1[C,c](=O)[N,n][O,o][C,c]1	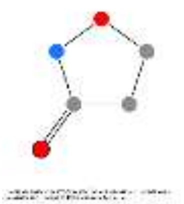	2
N-Oxyformamide in a ring	[C,c](=O)@[N,n]@[O,o]	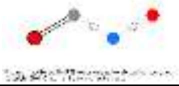	4
Five membered aromatic ring with a hydroxy group	[aR1]1[aR1][aR1](O([H]))[aR1][aR1]1	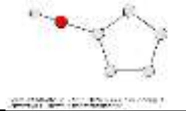	23
Five membered aromatic ring with a carbonyl group and an orto amino group	[aR1]1[aR1](N([H])([H]))[cR1](=O)[aR1][aR1]1	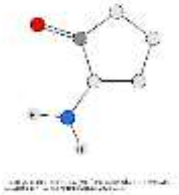	2

Information Classification: General

Table S5. Redox activity assays. Absorbance or fluorescence relative to the DMSO blank is reported. The threshold value for activity flag was greater than 10-fold the standard deviation over three duplicates of DMSO blank.

Label	HRP-PR assay (H_2O_2)			H_2DCFDA assay (ROS)			Resazurin assay (free radicals)		
	**Redox-free**	**1 mM DTT**	**Activity**	**Redox-free**	**100 µM TCEP**	**Activity**	**10 µM cpd**	**1 µM cpd**	**Activity**
**3**	1.1	1.0	Not active	1.1	1.0	Not active	1.0	1.1	Not active
**4**	0.8	1.0	Not active	3.4	1.9	Active	2.3	1.1	Active
**5**	0.9	1.0	Not active	3.9	2.4	Active	1.1	1.0	Not active
**6**	0.9	1.0	Not active	1.3	1.1	Not active	4.3	1.3	Active
**7**	0.9	1.0	Not active	1.4	2.1	Not active	1.1	1.0	Not active
**8**	0.8	1.0	Not active	8.0	10.6	Active	0.9	0.9	Not active

Table S6. Inhibitory properties of compound **3** against ATP on DdlB.

**Inhibition mechanism**	**Ki [µM]**	**R^2**
Competitive (full)	20.7 ± 4.5	0,90163
Competitive (partial)	20.7 ± 5.2	0,90163
Noncompetitive (full)	220.8 ± 21.7	0,86986

Table S7. Microbiological evaluation of fragment hit **3** and positive control D-cycloserine.

**Microorganism**	**MIC for 3**	**MIC for D-cycloserine**
E. coli ATCC 25922	> 128 µg/mL	64 µg/mL
S. aureus ATCC 29213	> 128 µg/mL	32 µg/mL
E. coli N43	> 128 µg/mL	32 µg/mL
E. coli D22	> 128 µg/mL	16 µg/mL

Information Classification: General

2 Supplemental figures

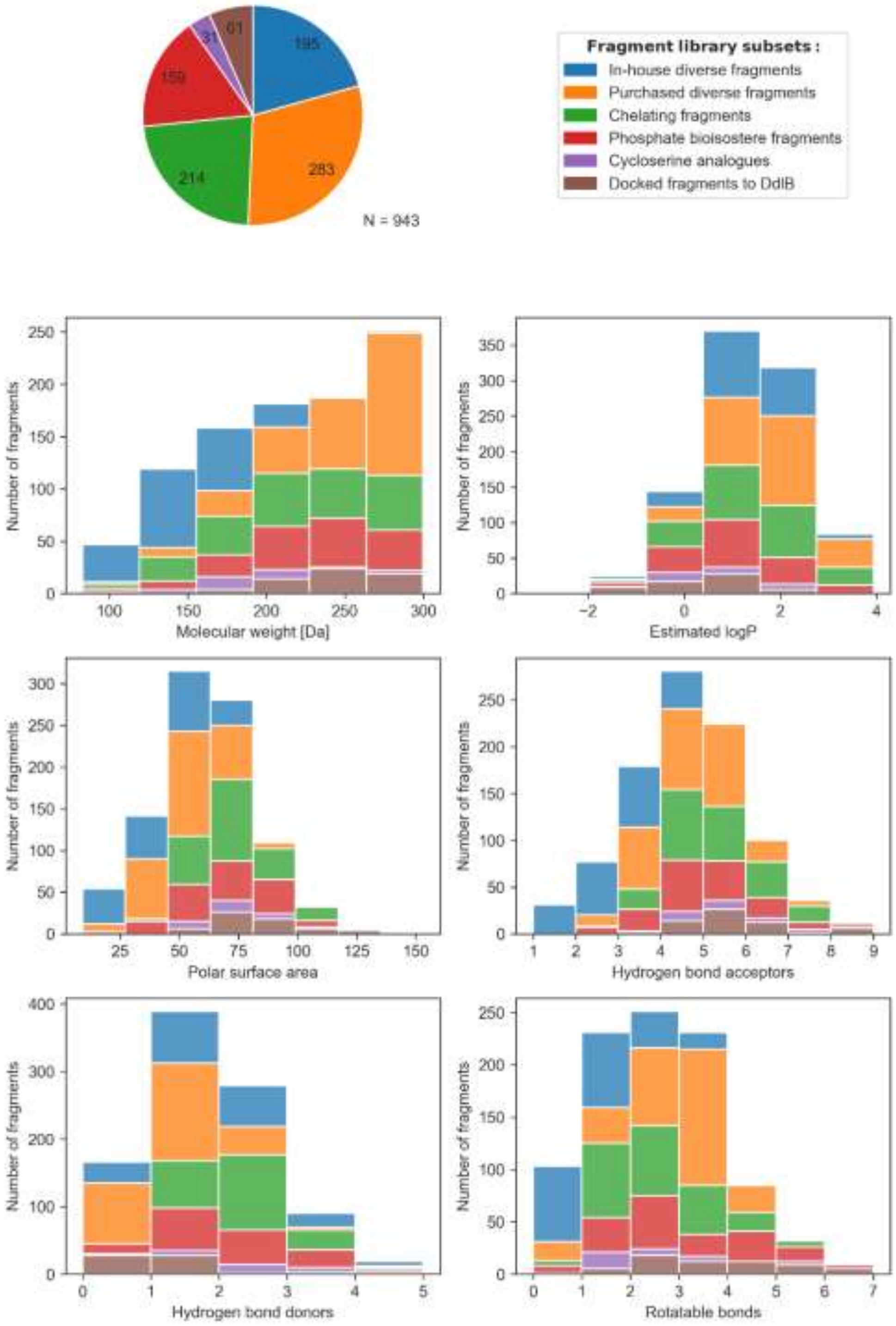

Figure S1. Number of fragments in each of the fragment library subsets and the distribution of selected physicochemical properties.

Information Classification: General

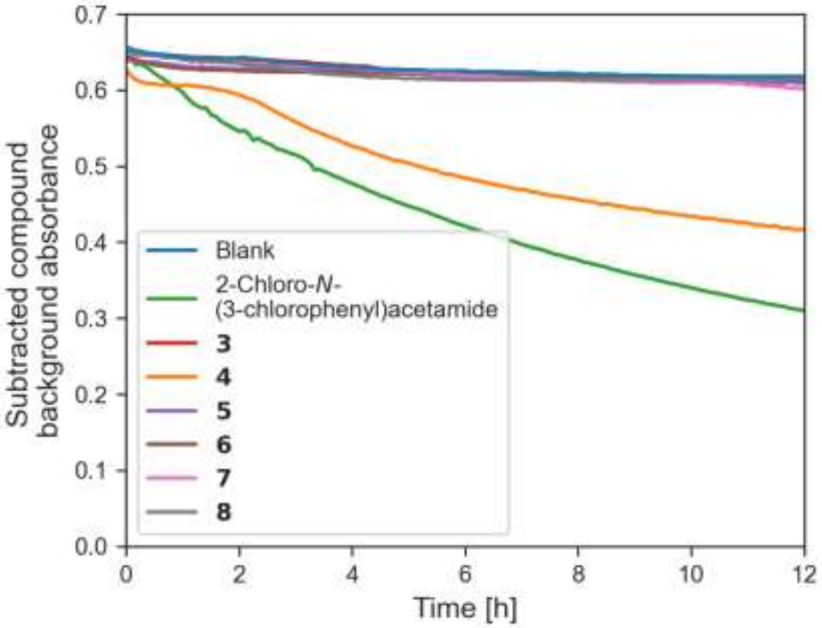

Figure S2. Thiol reactivity assay for hits from the fragment screening campaign.

Information Classification: General

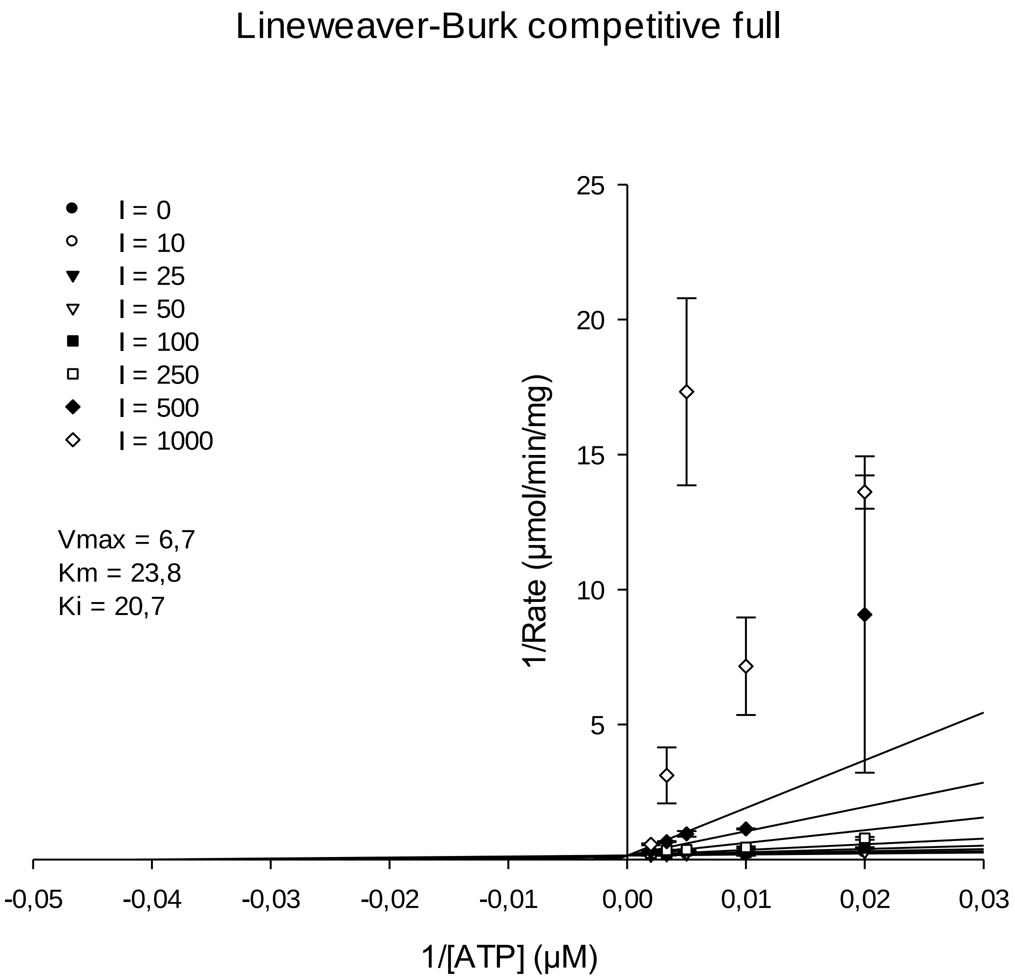

Figure S3. DdlB inhibition kinetics for compound **3**.

Information Classification: General

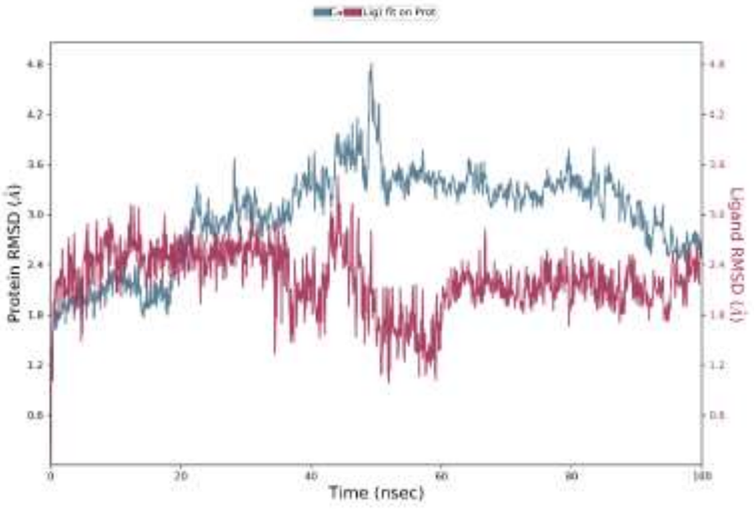

Figure S4. The RMSD plot over simulation time – the teal-coloured plot corresponds to the DdlB Cα RMSD and the red-coloured to ligand RMSD. For calculation of both parameters, all frames were first aligned to the backbone in the first, reference frame.

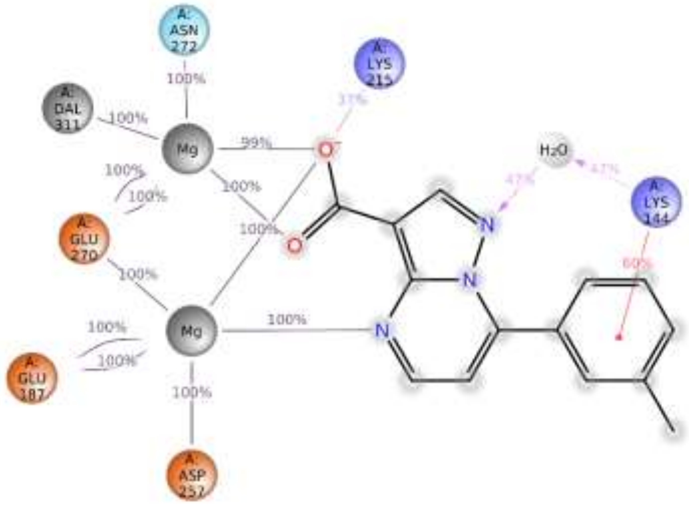

Figure S5. A ligand interaction diagram for a 100 ns MD simulation of **3** in DdlB. The protein-ligand contacts and interactions that occur for more than 30% of the MD simulation time are shown: the metal coordination as grey lines, the cation–π interactions as red lines, hydrogen bonds are shown in blue, and ionic interactions in magenta. Grey circles denote solvent exposure.

Information Classification: General

3 HPLC traces

3

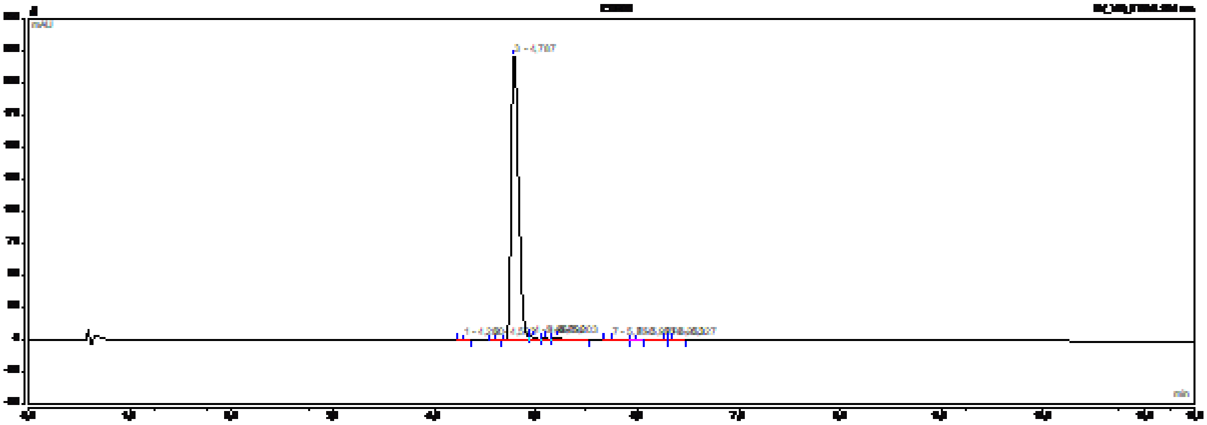

6

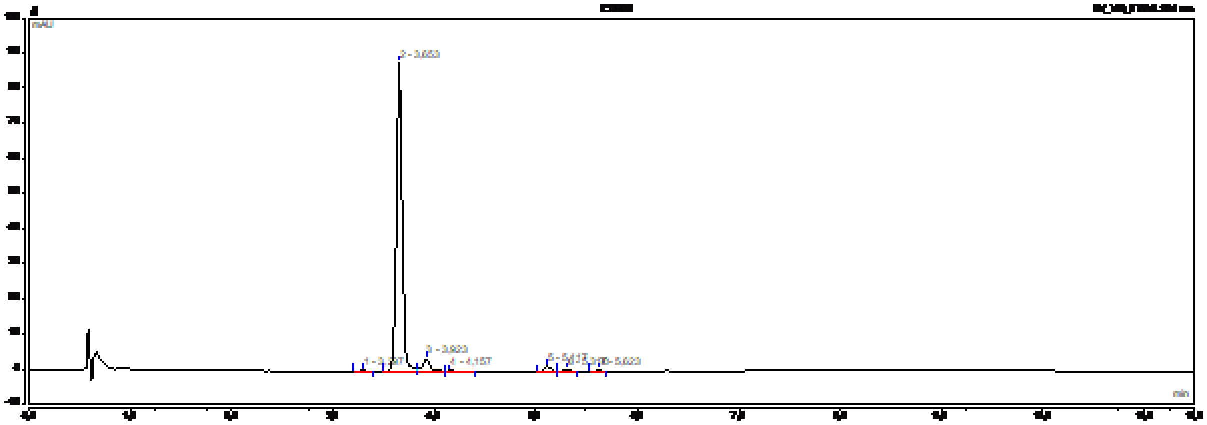

7

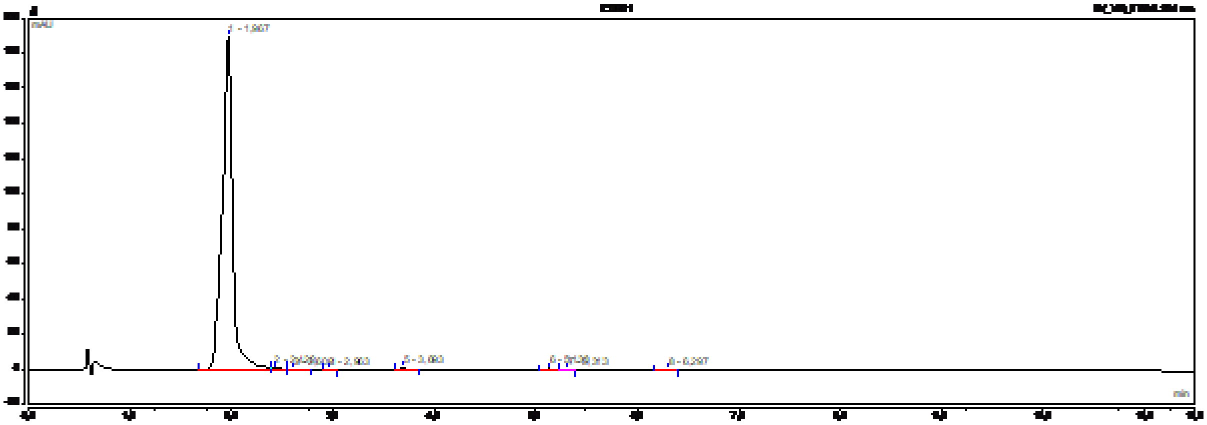

Information Classification: General

9

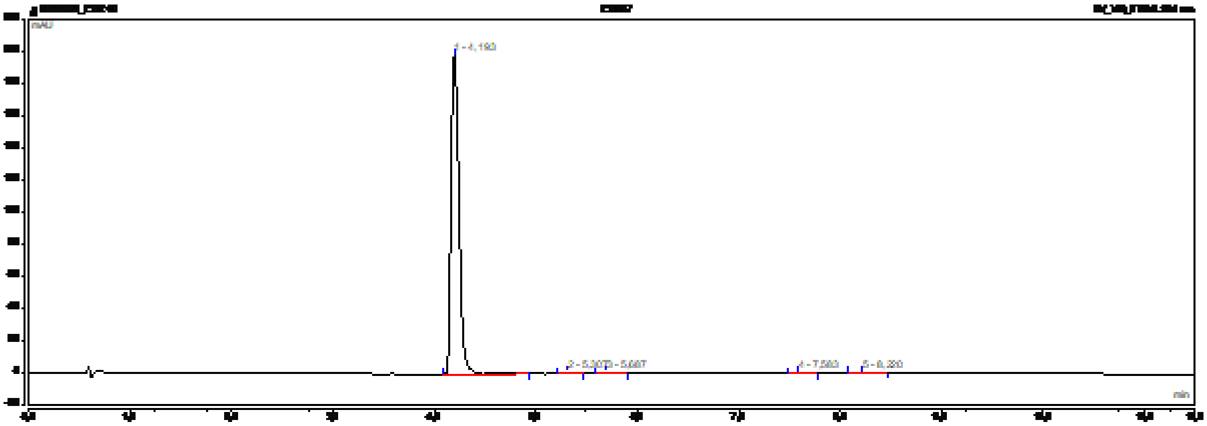

10

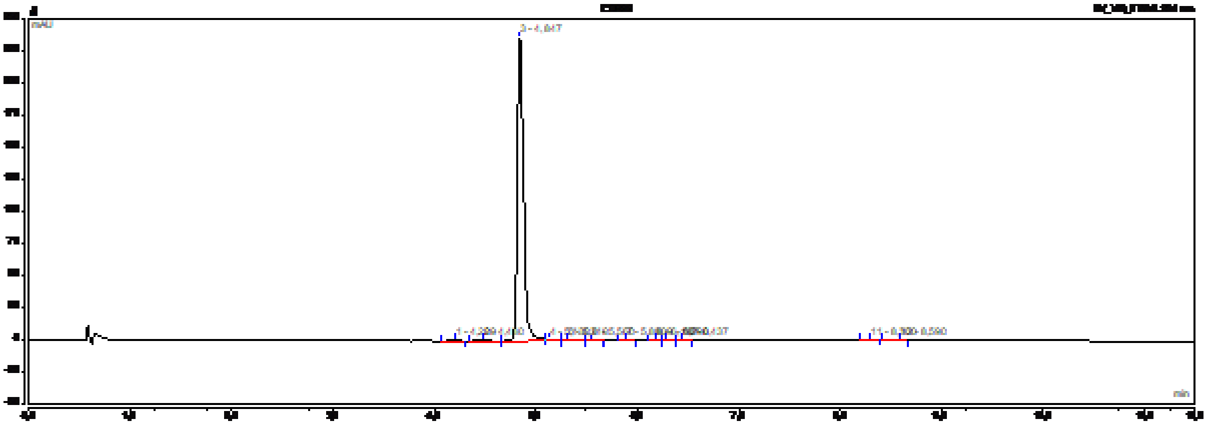

11

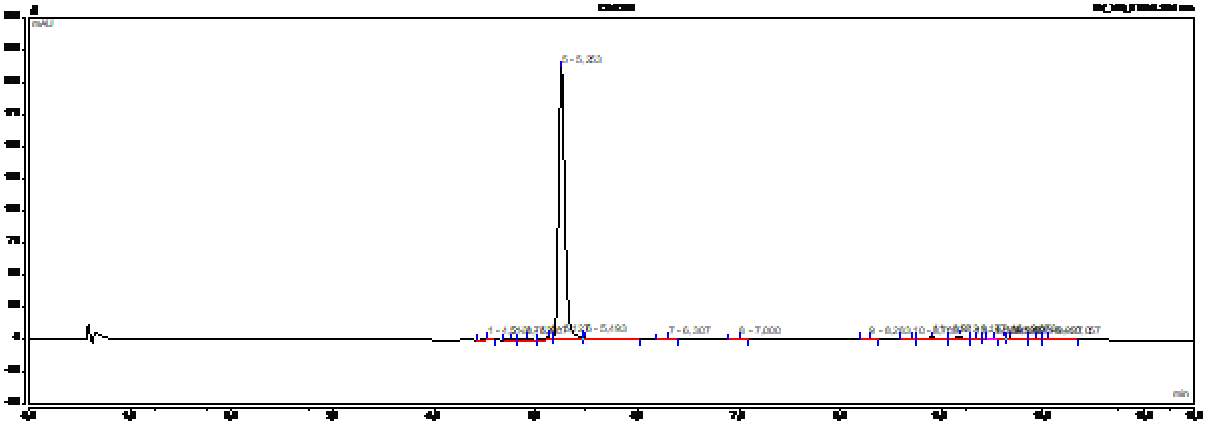

Information Classification: General

12

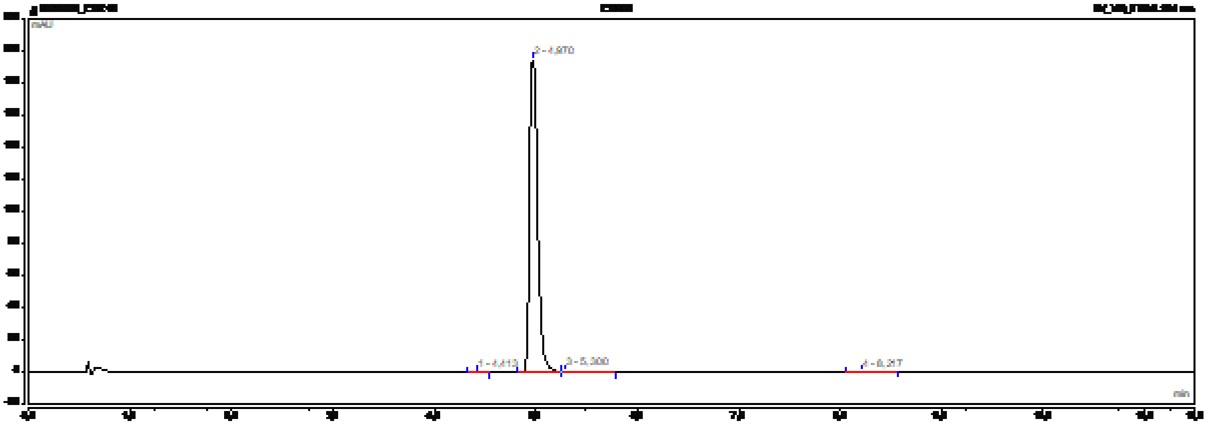

13

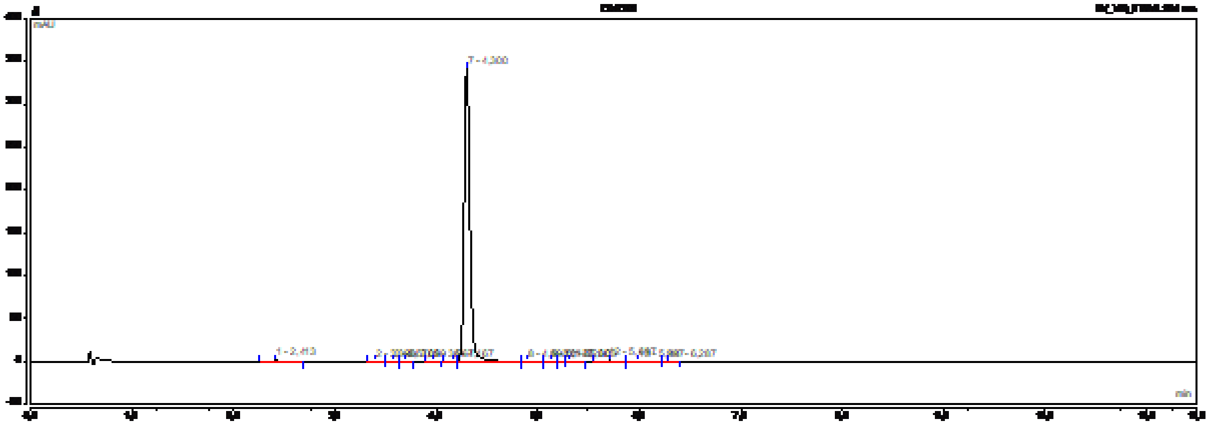

14

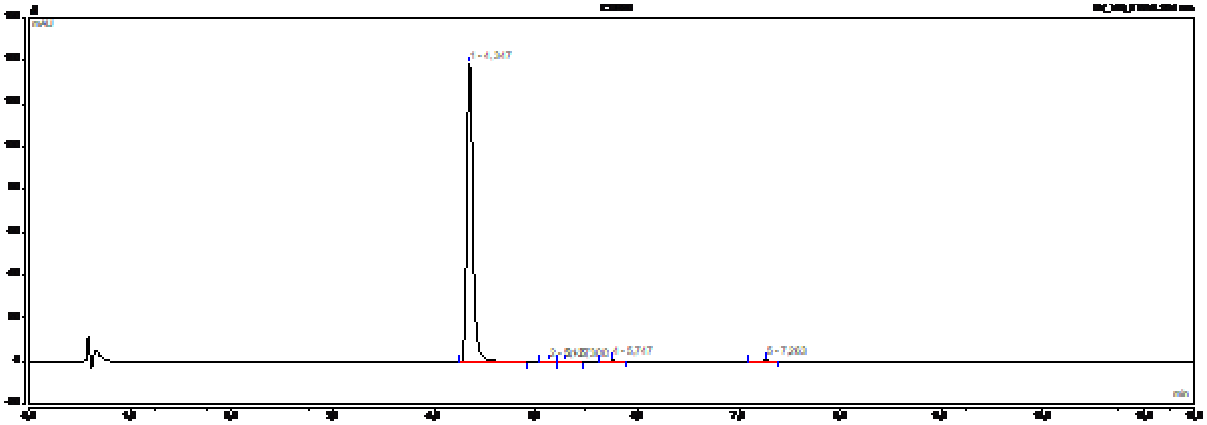

Information Classification: General

15

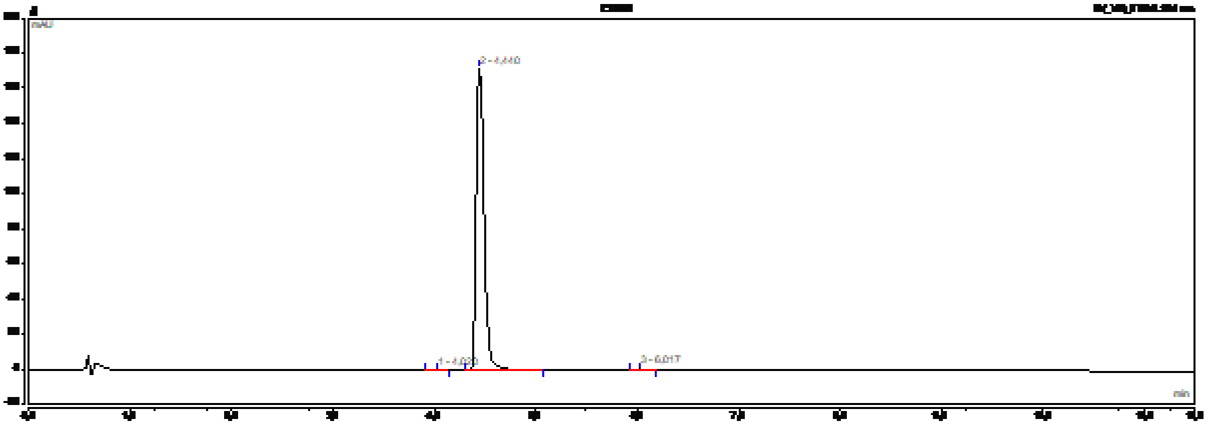

16

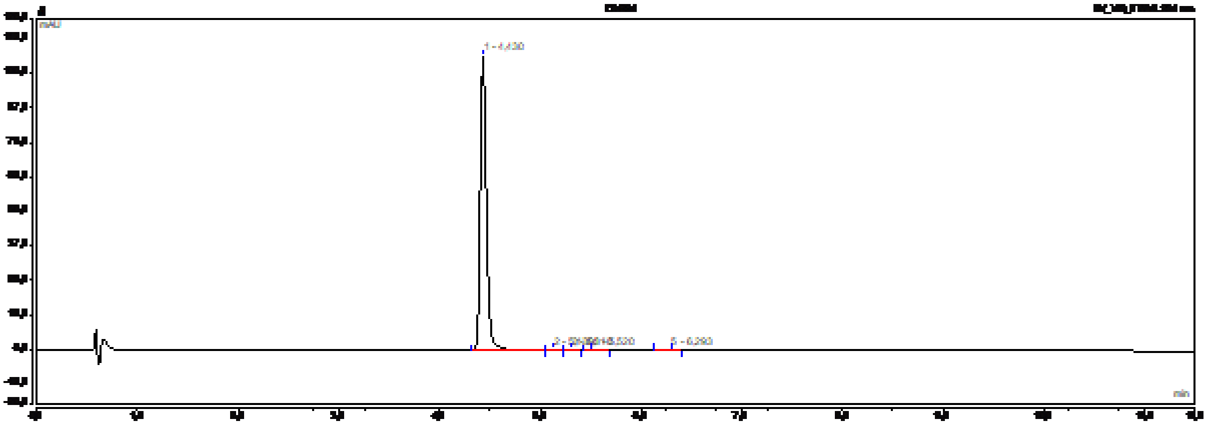

17

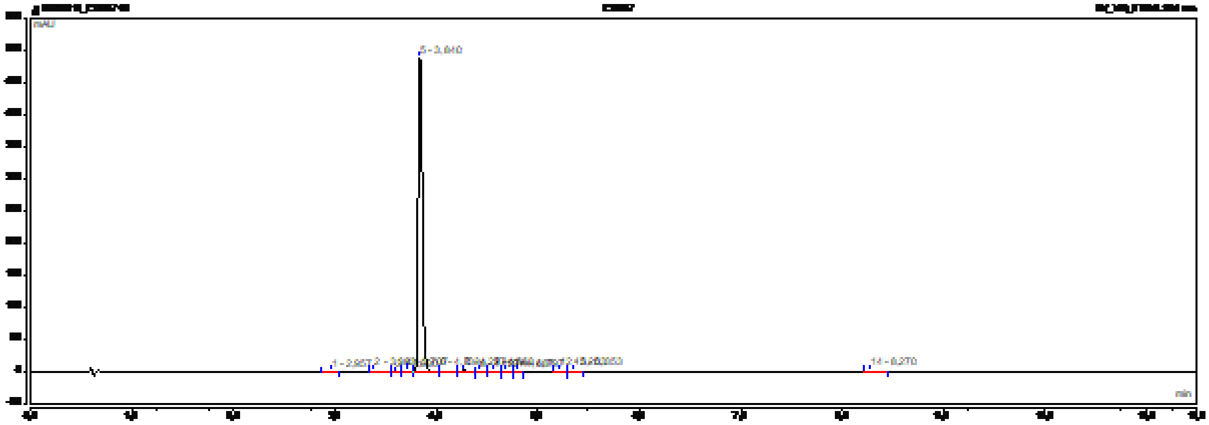

Information Classification: General

18

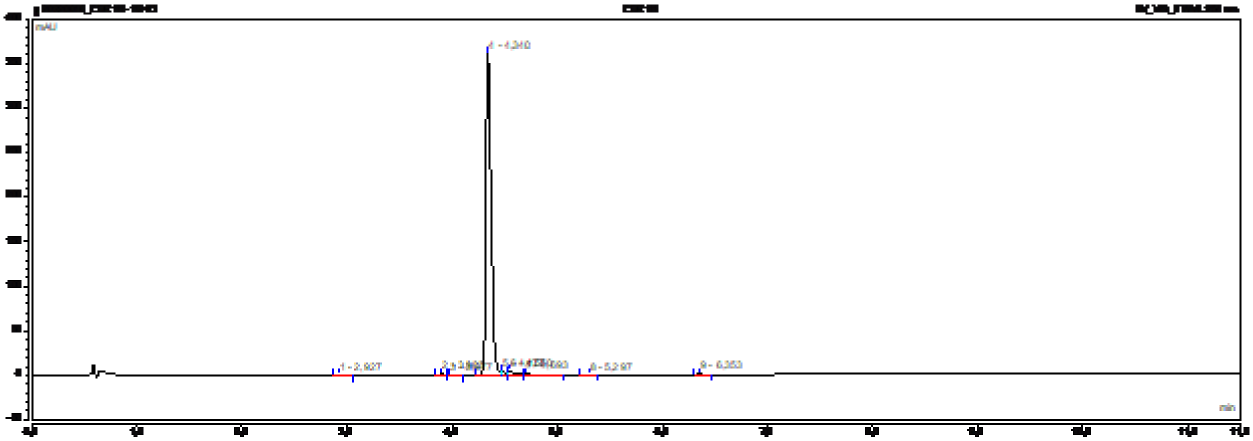

19

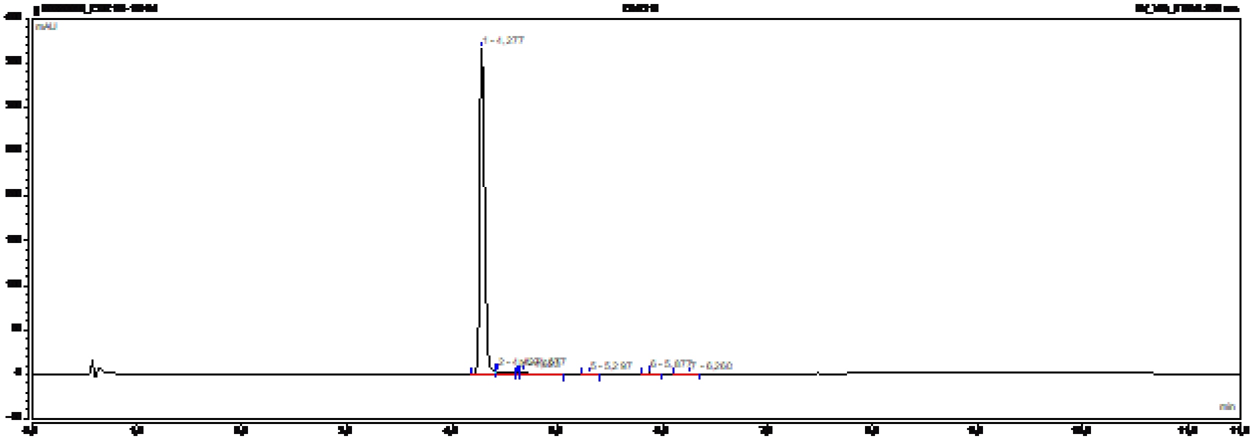

20

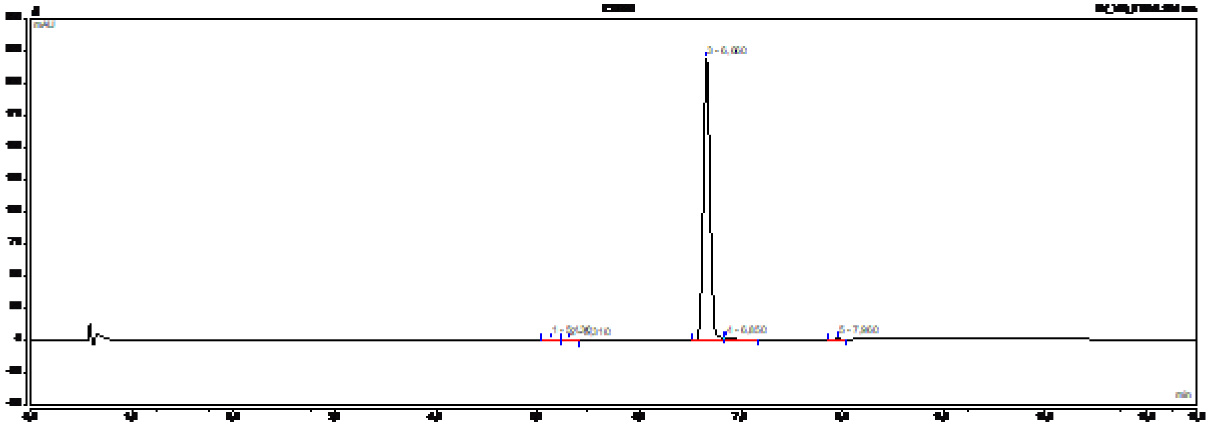

Information Classification: General

21

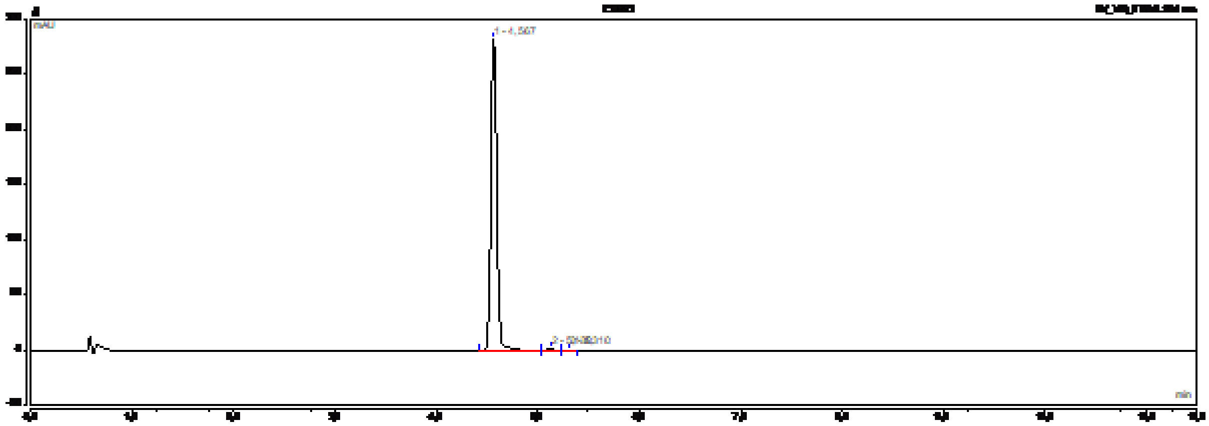

22

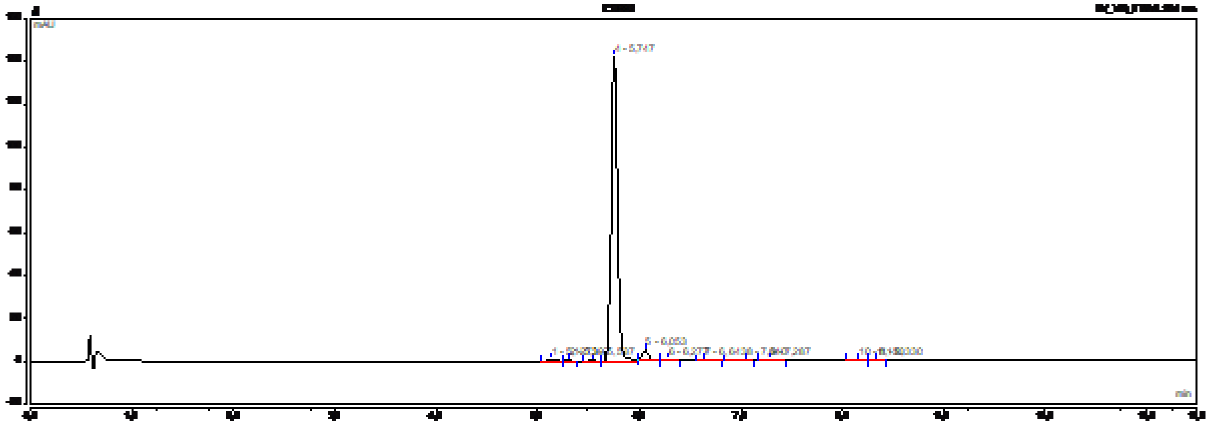

23

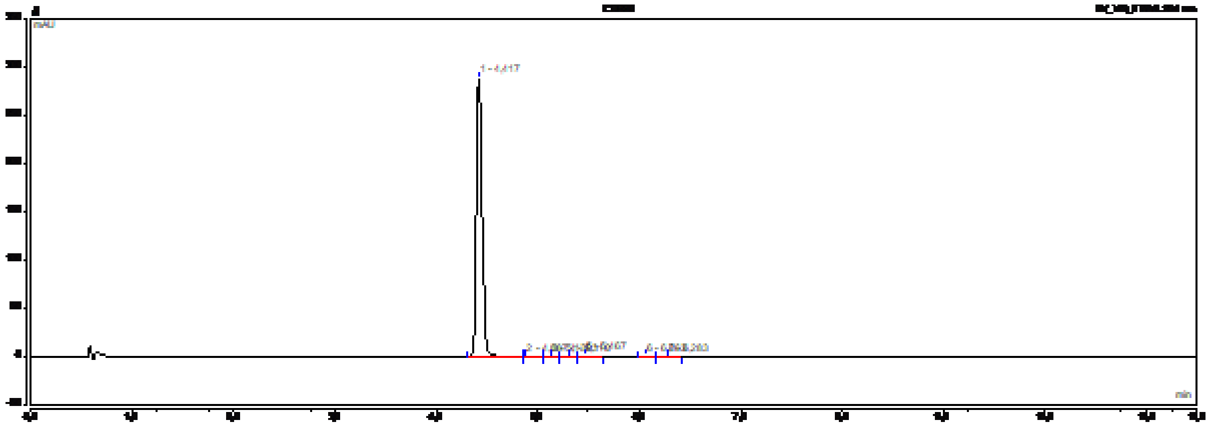

Information Classification: General

4 NMR characterization

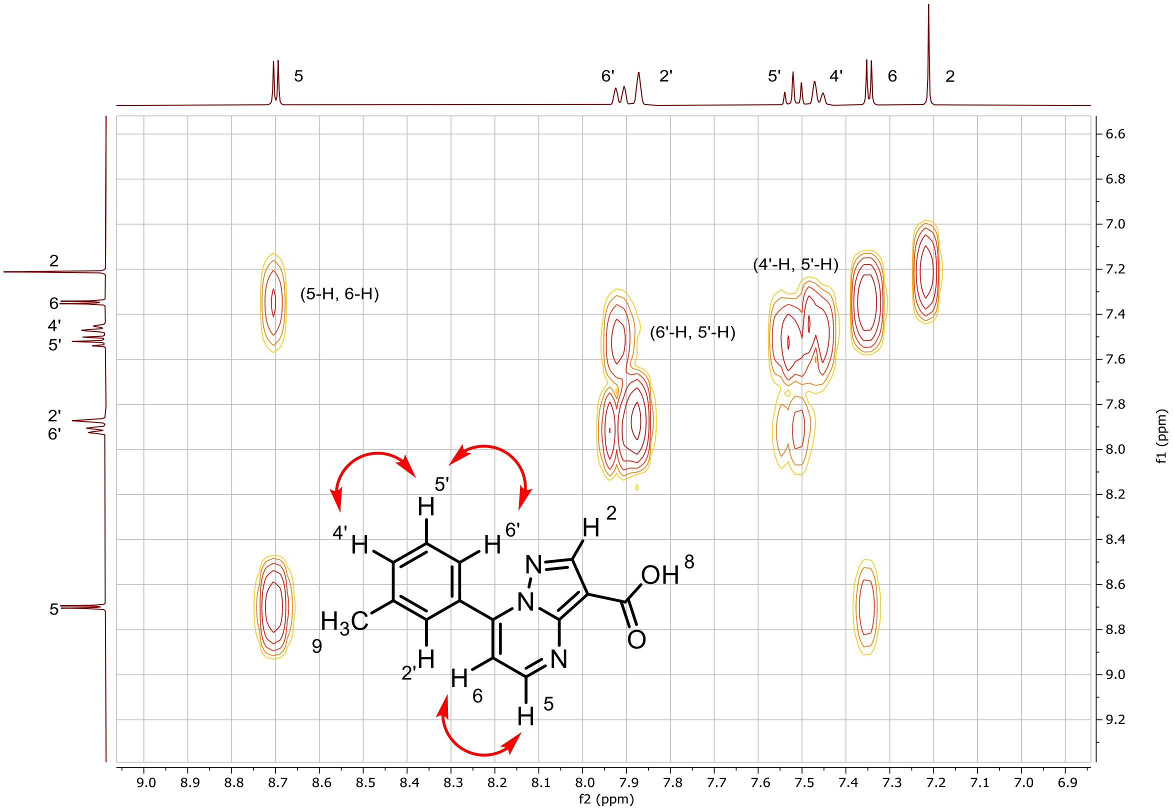

Figure S6. ^{1}H-^{1}H COSY experiment for **3**, DMSO-d_6.

Information Classification: General

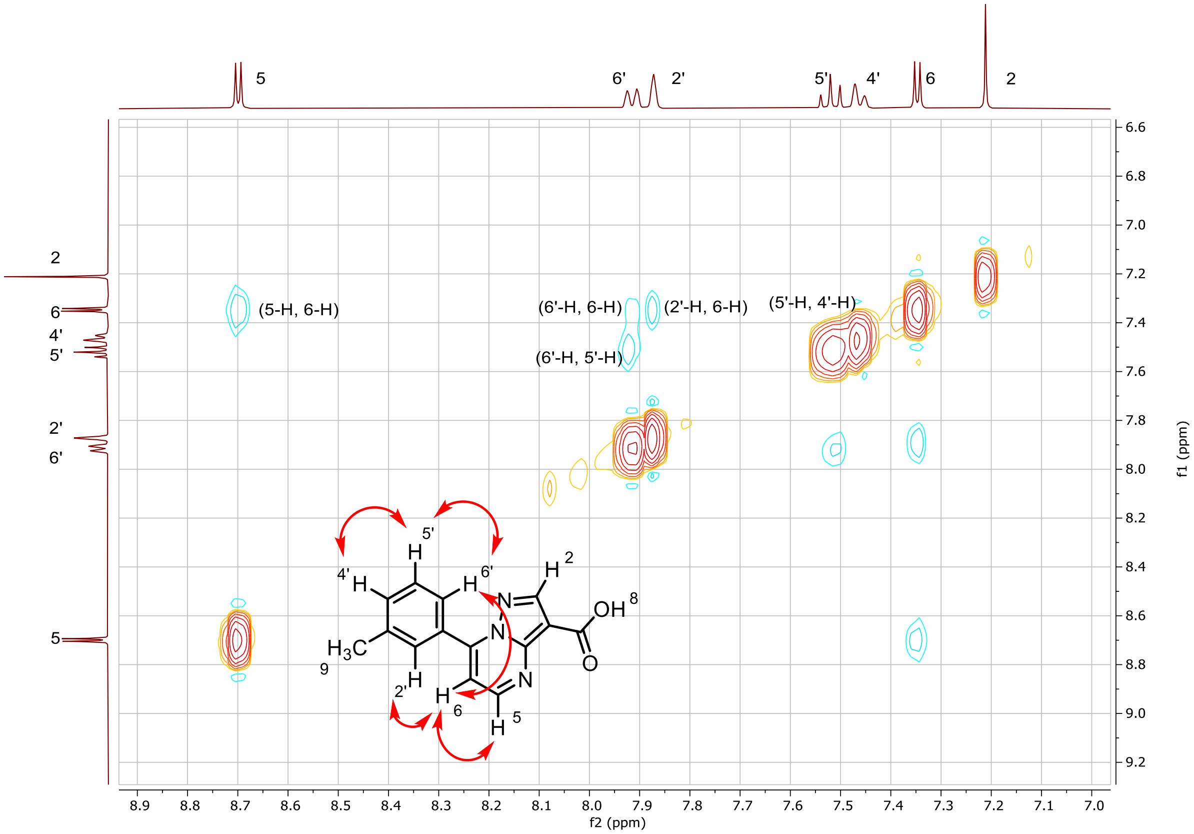

Figure S7. ^{1}H-^{1}H NOESY experiment for **3**, DMSO-d_6.

Information Classification: General

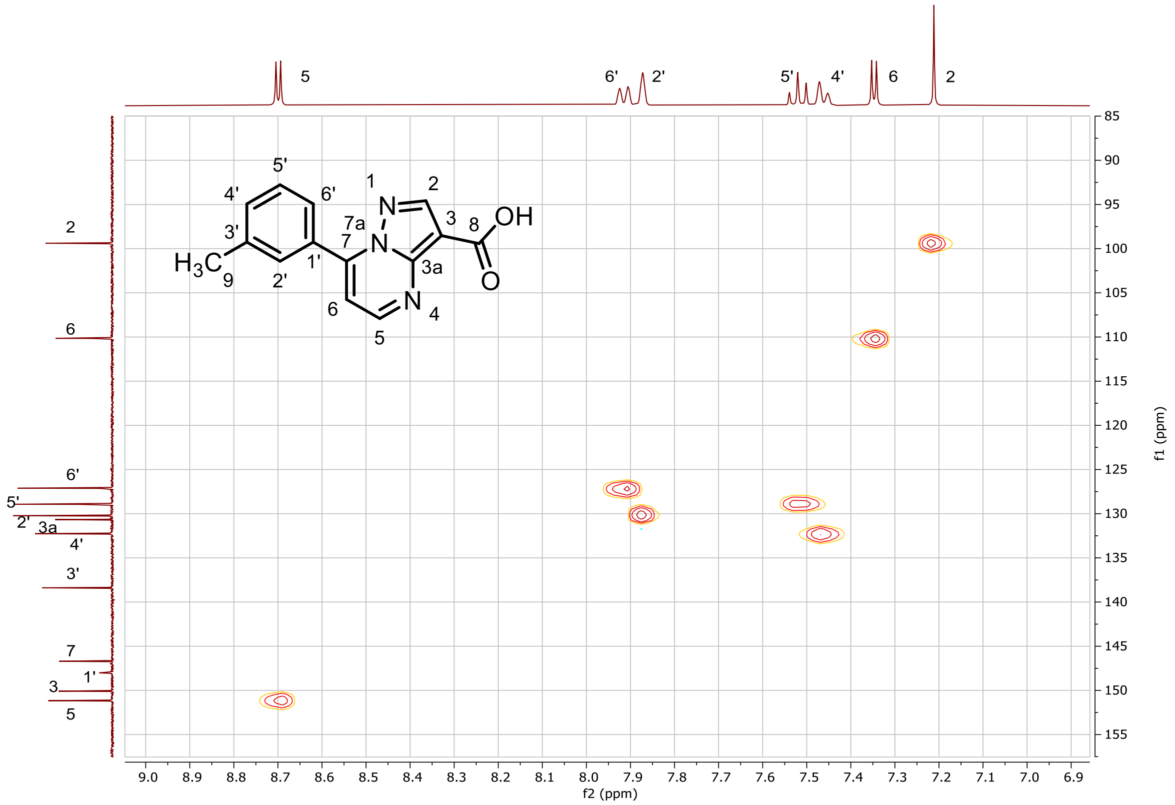

Figure S8. ^{1}H-^{13}C HSQC experiment for **3**, DMSO-d_6.

Information Classification: General

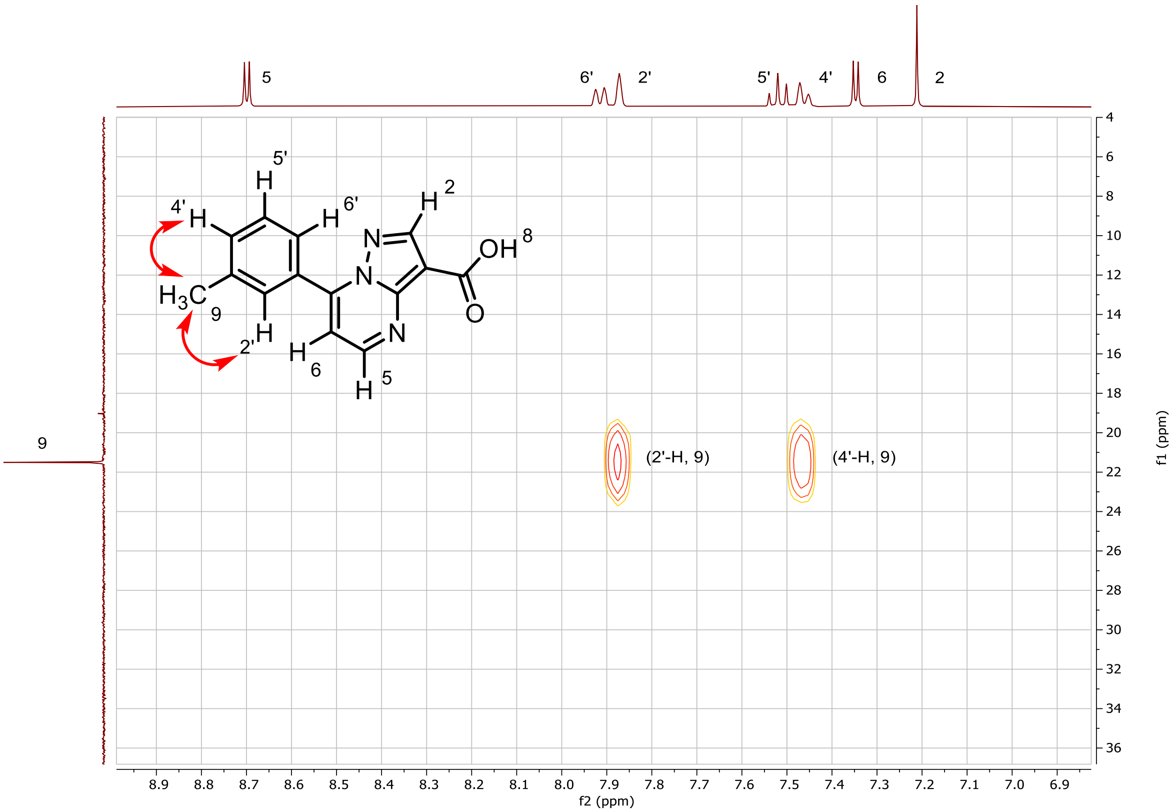

Figure S9. ^{1}H-^{13}C HMBC experiment for **3**, DMSO-*d*$_6$. Cross-peaks for CH_3 carbon are shown.

Information Classification: General

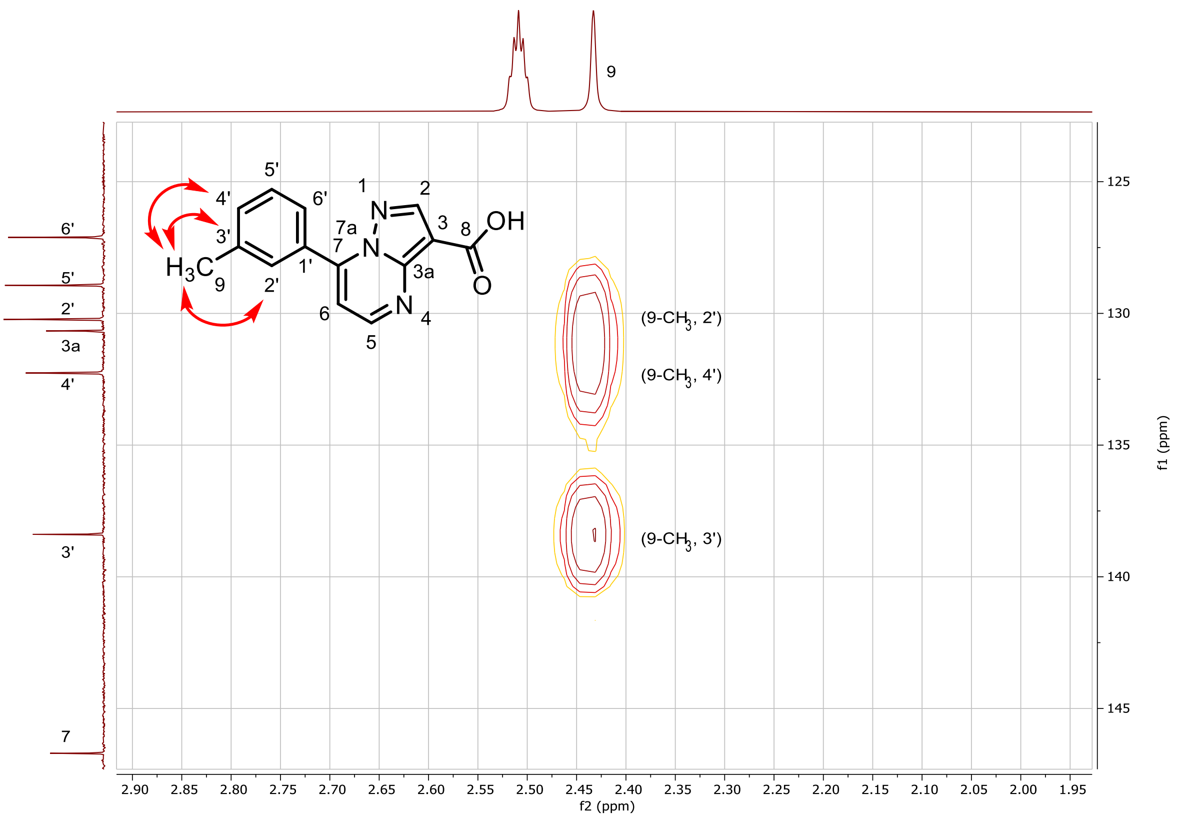

Figure S10. ^{1}H-^{13}C HMBC experiment for **3**, DMSO-d_6. Cross-peaks for CH_3 protons are shown.

Information Classification: General

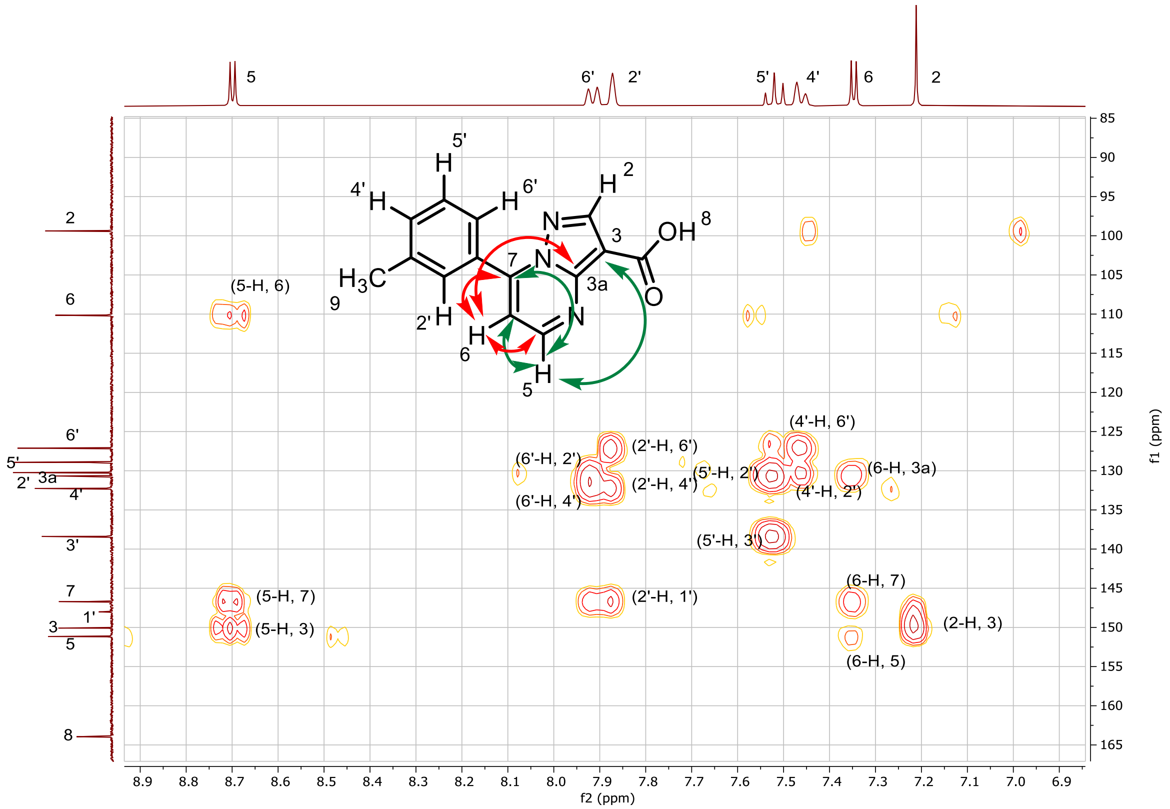

Figure S11. ^{1}H-^{13}C HMBC experiment for **3**, DMSO-d_6. Cross-peaks for the aromatic carbons and protons are shown.

Information Classification: General

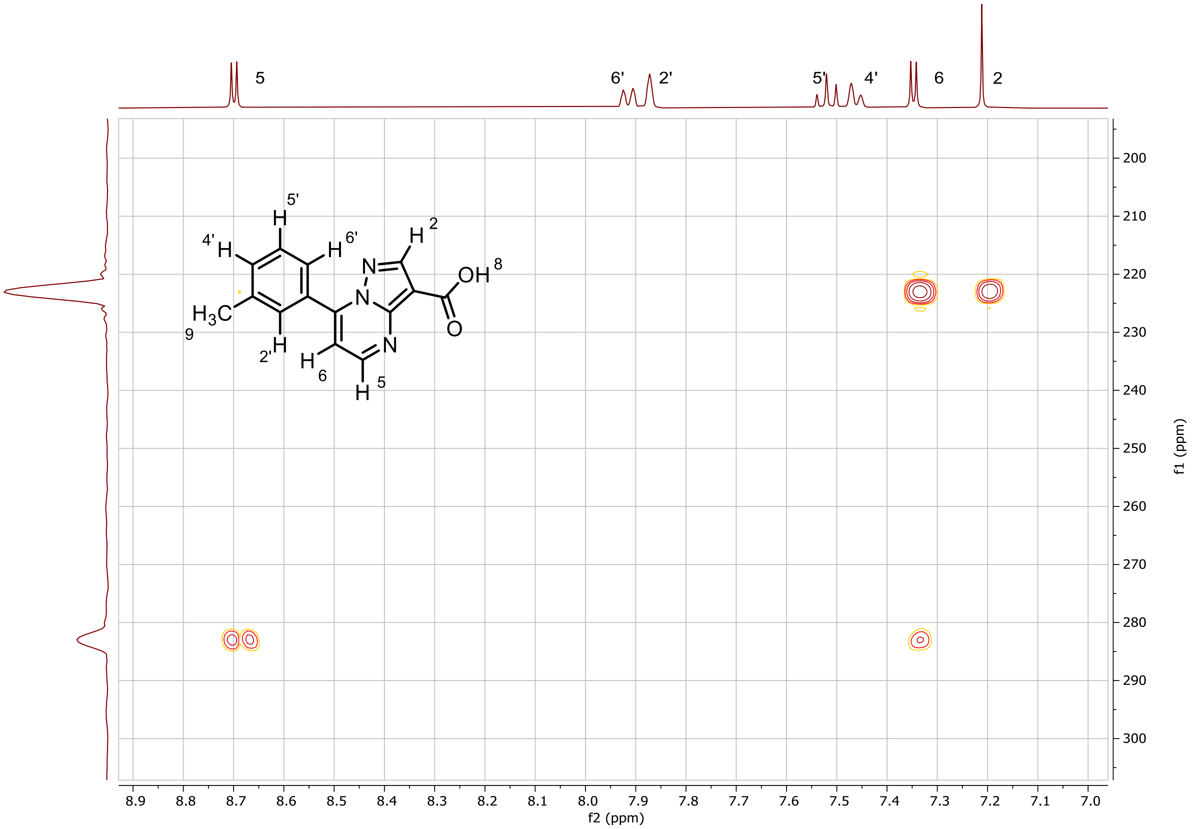

Figure S12. ^{1}H-^{15}N HMBC experiment for **3**, DMSO-d_6.

5 Supplemental references

[1] Brenk, R.; Schipani, A.; James, D.; Krasowski, A.; Gilbert, I. H.; Frearson, J.; Wyatt, P. G. Lessons Learnt from Assembling Screening Libraries for Drug Discovery for Neglected Diseases. *ChemMedChem*, **2008**, *3* (3), 435–444. https://doi.org/10.1002/cmdc.200700139.

Information Classification: General
